# Supplementary material for: Clodronate Improves Survival of Transplanted Hoxb8 Myeloid Progenitors with Constitutively Active GMCSFR in Immunocompetent Mice
Source: Mol Ther Methods Clin Dev. 2017 Sep 7;7:60–73. doi: 10.1016/j.omtm.2017.08.007 (PMC5633862; doi:10.1016/j.omtm.2017.08.007)
Supplement: Document S1. Supplemental Materials and Methods and Figures S1–S9 [file mmc1.pdf]

OMTM, Volume 7

Supplemental Information

Clodronate Improves Survival of Transplanted Hoxb8 Myeloid Progenitors with Constitutively Active GMCSFR in Immunocompetent Mice

Simon Lee, Saul Kivimäe, and Francis C. Szoka

1 Supplemental Figures

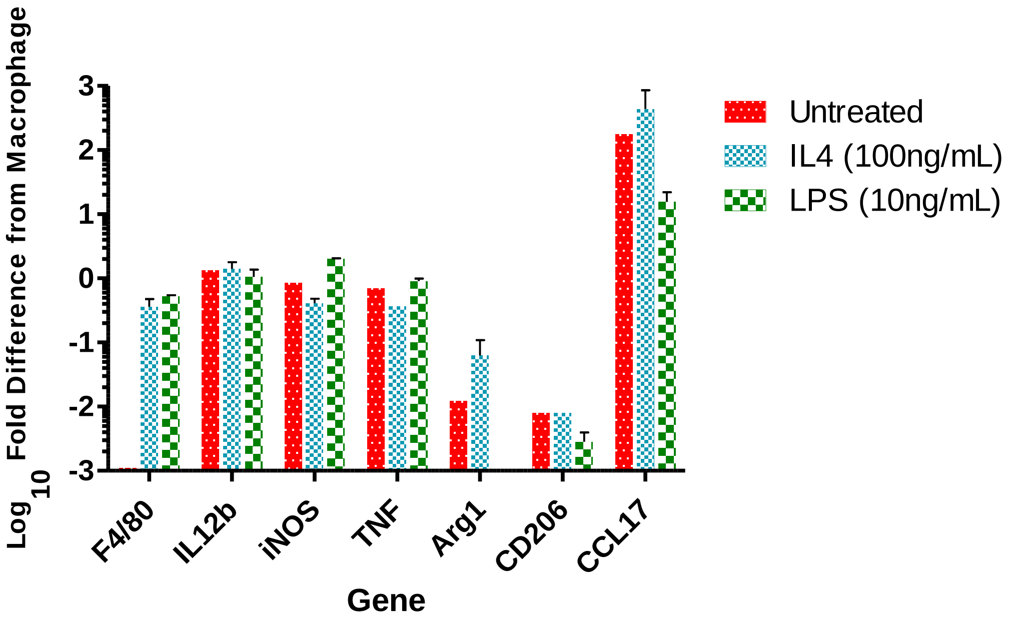

Figure S1: Gene expression of HDP-on treated with M1 and M2 inducers. HDP-on were treated overnight with 10 ng/mL LPS or 100 ng/mL IL-4 and gene expression of established M1 and M2 genes (M1: IL12b, iNOS, TNF; M2: Arg1, CD206, CCL17) was measured by qPCR. Fold enhancement is expressed relative to untreated HDP-on MΦs.

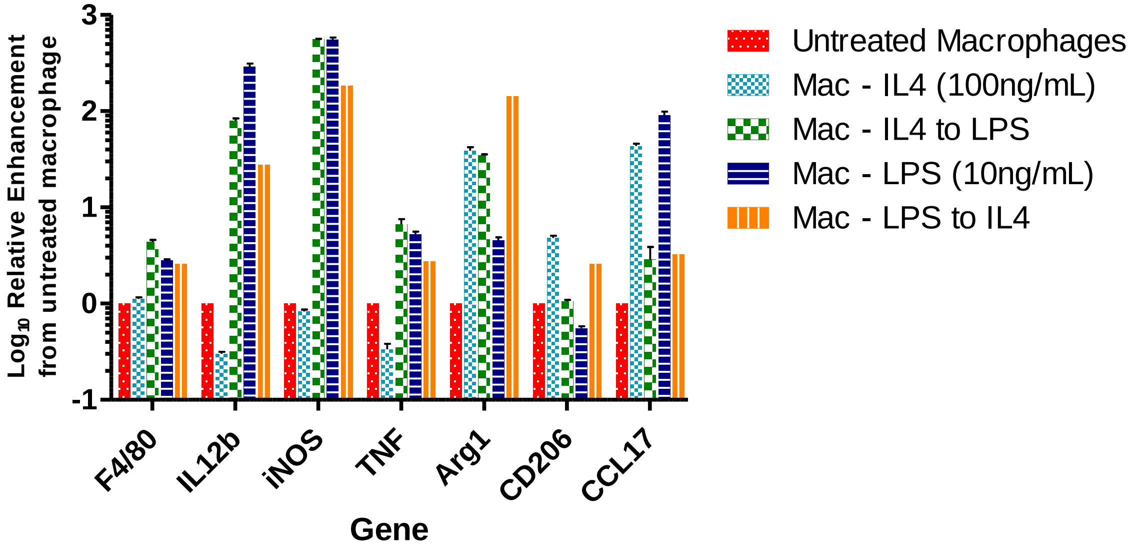

Figure S2: Relative gene expression of MΦ and M1/M2 markers of M1/M2 polarized HDP-on MΦ treated with opposing M1/M2 inducers. Cultures were treated for 24 h with 100 ng/mL IL-4 or 10 ng/mL LPS. For IL-4 to LPS or LPS to IL-4 cultures, cells were treated for 24 h with 100 ng/mL IL-4 or 10 ng/mL LPS before the media was swapped for the opposing treatment for another 24 h. MΦ were differentiated for 6 days in 40 nM 4-OHT and 1 μM ruxolitinib in tissue culture plastic flasks before $1x10^5$ cells were plated in a 12 well plate overnight before treatment. Relative expression is calculated by comparing expression levels between differentiated cells and HDP-on MΦ.

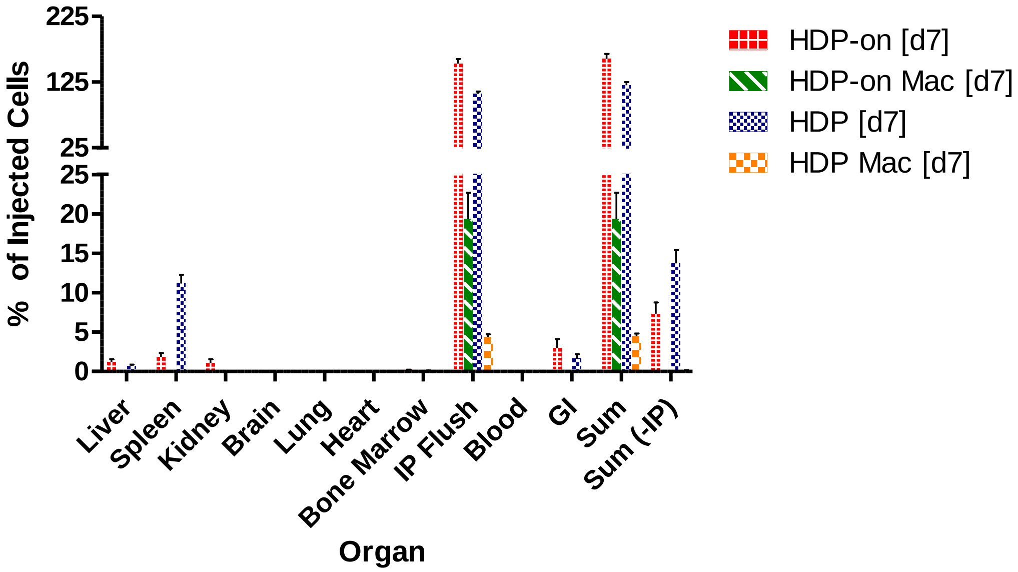

Figure S3: Biodistribution of HDP, HDP-on, HDP MΦs and HDP-on MΦs in immunodeficient NCG mice 7 days post injection. Mice were injected intraperitoneally with $5x10^6$ cells in 500 μL RPMI with a 28 gauge syringe, euthanized after 7 days and tissues were analyzed for luciferase activity. Statistics: N = 6 for HDP-on, N = 3 for other conditions.

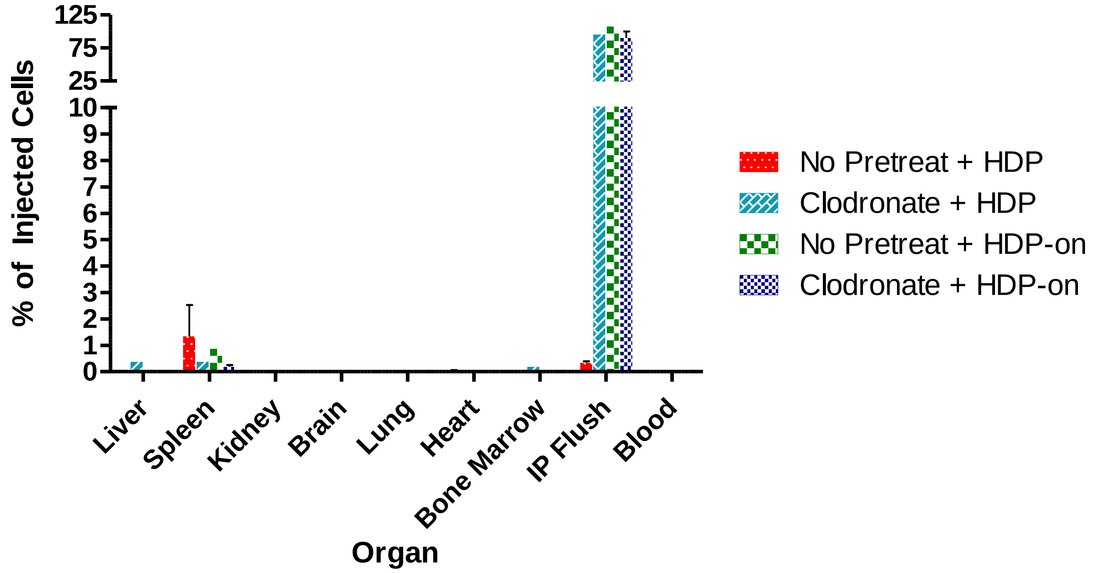

Figure S4: Biodistribution of HDP and HDP-on in mice pretreated with liposomal clodronate 1 day post-transplantation. For clodronate pretreatment, mice were injected IP with 100 µL liposomal clodronate (5mg/mL) 4 and 1 days before cell injection. Healthy BALB/c mice were injected intraperitoneally with $5x10^6$ cells in 500 µL RPMI with a 28 gauge syringe, euthanized after 1 days and tissues were analyzed for luciferase activity. Statistics: N = 3 per condition

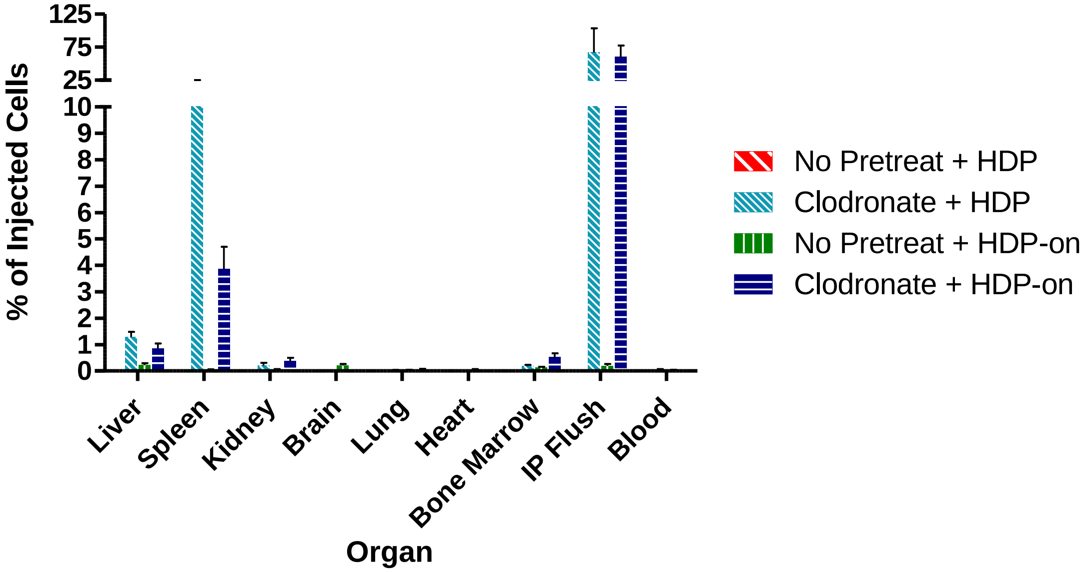

Figure S5: Biodistribution of HDP and HDP-on in mice pretreated with liposomal clodronate 7 days post-transplantation. For clodronate pretreatment, mice were injected IP with 100 µL liposomal clodronate (5mg/mL) 4 and 1 days before cell injection. Healthy BALB/c mice were injected intraperitoneally with $5x10^6$ cells in 500 µL RPMI with a 28

gauge syringe, euthanized after 7 days and tissues were analyzed for luciferase activity. Statistics: N = 3 for HDP with no liposomal clodronate, N = 6 for all other conditions

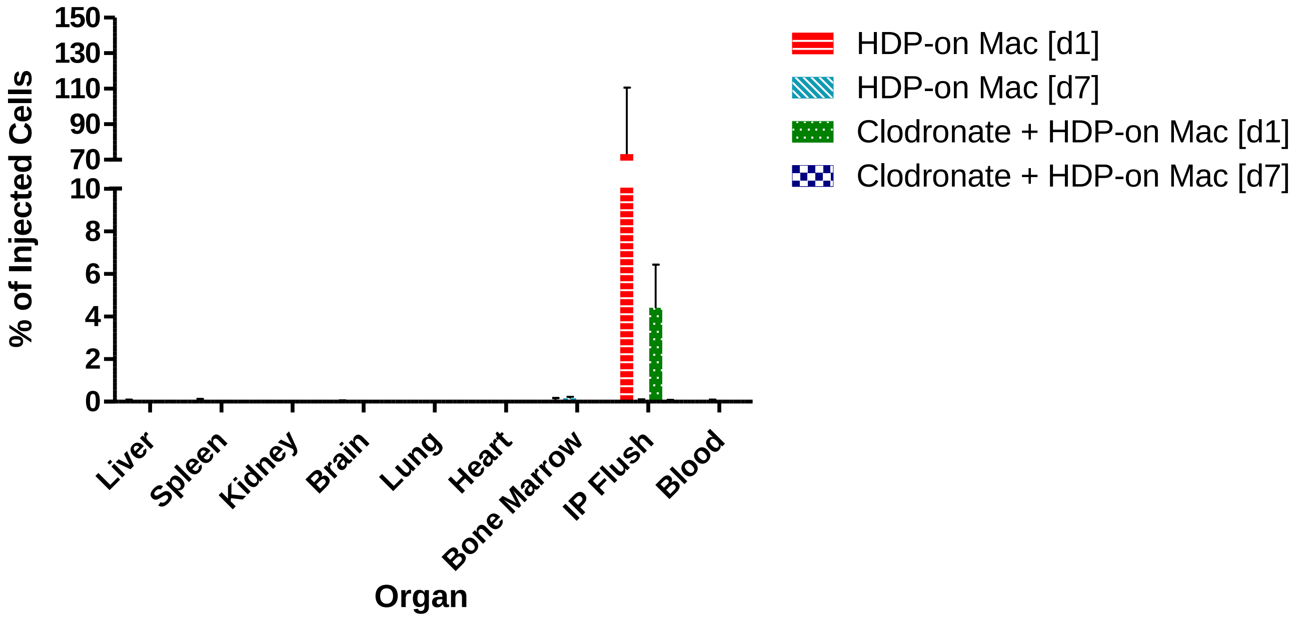

Figure S6: Biodistribution of macrophages derived from HDP and HDP-on in mice pretreated with liposomal clodronate 1 and 7 days post-transplantation. Healthy BALB/c mice were injected intraperitoneally with $5x10^6$ cells in 500 µL RPMI with a 28 gauge syringe, euthanized after 1 or 7 days and tissues were analyzed for luciferase activity. Statistics: N = 3 for all conditions

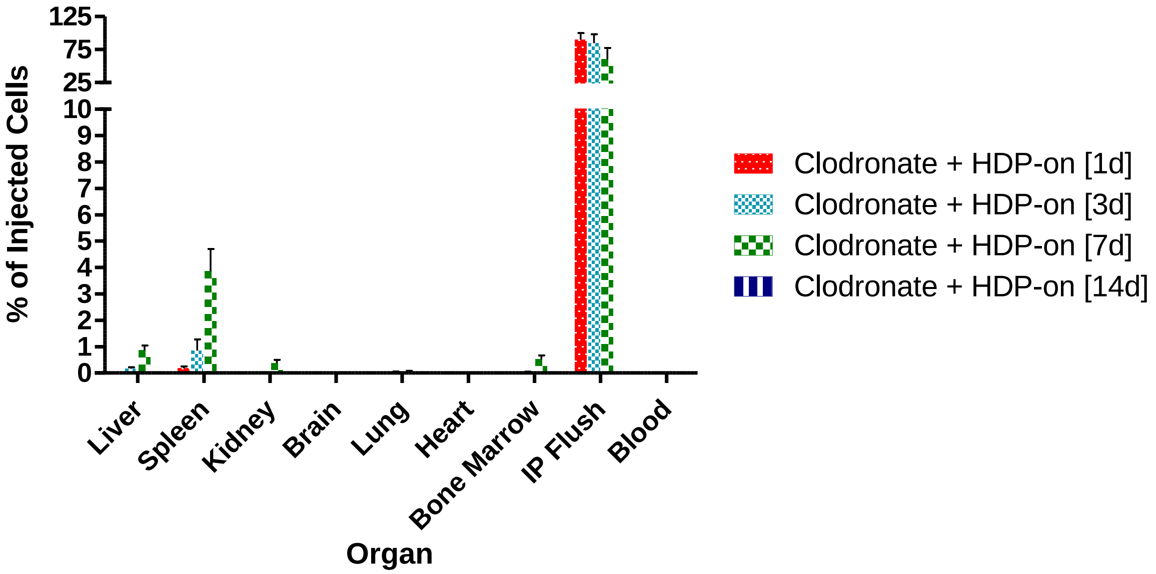

Figure S7: Biodistribution of HDP-on in clodronate-pretreated BALB/c mice at 1, 3, 7 and 14 days post-transplantation. Mice were injected IP with 100 µL liposomal clodronate (5mg/mL) 4 and 1 days before cell injection. BALB/c mice were injected intraperitoneally

with $5x10^6$ cells in 500 µL RPMI with a 28 gauge syringe, euthanized after 14 days and tissues were analyzed for luciferase activity. Statistics: N = 3 per time point

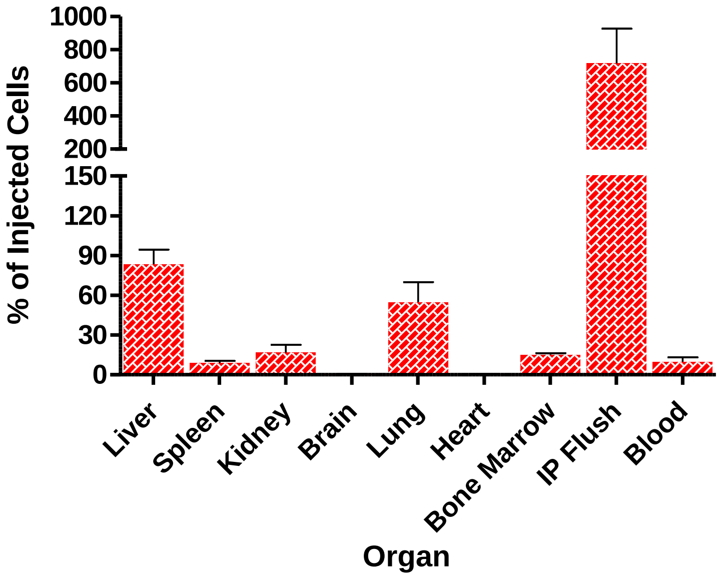

Figure S8: Biodistribution of HDP-on in immunodeficient NCG mice 14 days post-transplantation. NCG mice were injected intraperitoneally with $5x10^6$ cells in 500 µL RPMI with a 28 gauge syringe, euthanized after 14 days and tissues were analyzed for luciferase activity. Statistics: N = 3

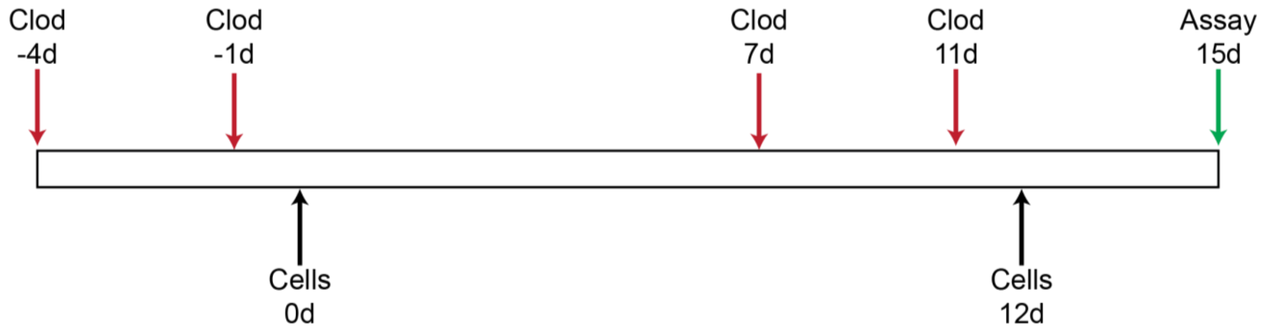

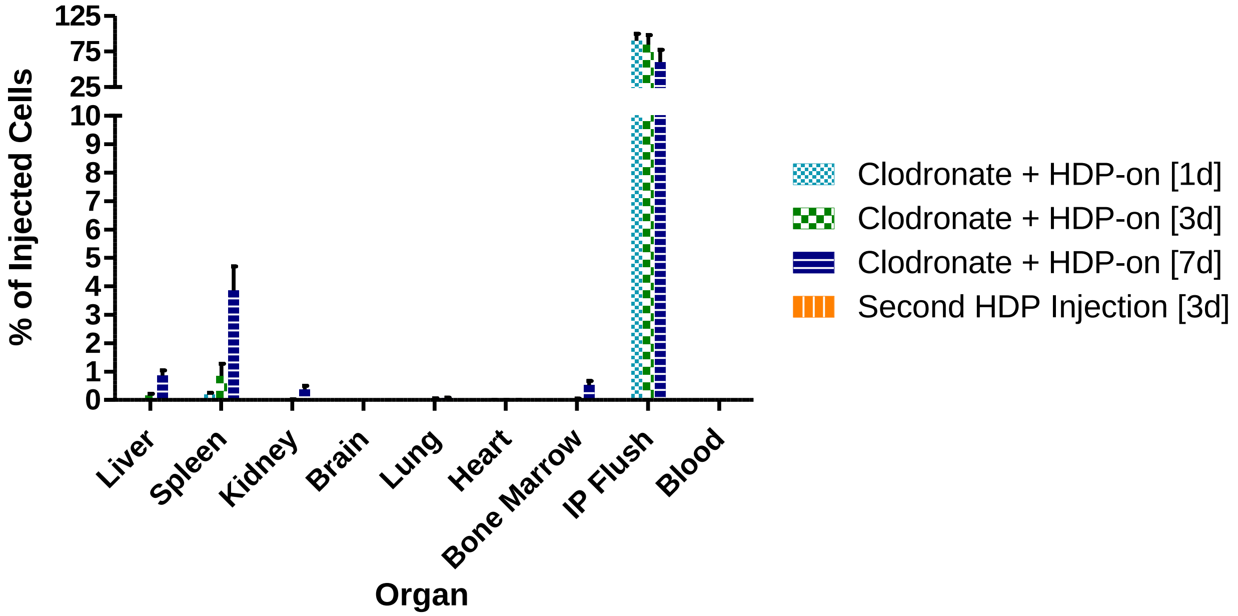

Figure S9: Biodistribution of HDP-on in clodronate-pretreated BALB/c mice which received a second injection of HDP-on. (Top): Treatment scheme of mice which received second injection. (Bottom): Biodistribution of mice which received second injection, presented with data from mice which receive only a single injection of HDP-on. Mice were injected IP with 100 µL liposomal clodronate (5 mg/mL) 4 and 1 days before cell injection. BALB/c mice were injected intraperitoneally with $5x10^6$ cells in 500 µL RPMI with a 28 gauge syringe, euthanized after 14 days and tissues were analyzed for luciferase activity. Statistics: N = 3 per time point

2 Methods

2.1 Supplemental GM-CSF Media

A GM-CSF expressing L929 cell line was generated by transducing L929 cells with a lentiviral construct (pLVX-GMCSF-IRES-tdTomato). Positively transduced cells were isolated by FACS and used to generate the supplemental media. GM-CSF L929 cells were grown to confluency, whereupon the media was switched to a low serum formulation (DMEM, 50 mM HEPES, 1% PenStrep/Amphotericin B antibiotic/antimycotic, 1% Glutamax (Gibco), 0.5% heat inactivated fetal calf serum (Hyclone)). This reduces the proliferation capacity of the cells but maintains survival. After 3 days, the media was collected, centrifuged, filtered and frozen for future use. GM-CSF levels were measured following the protocols from a GM-CSF ELISA kit from Boster.

2.2 Lin^- bone marrow culture and infection

Preparation:

Progenitor outgrowth media:

RPMI + 10% FBS + 50 µM 2-mercaptoethanol + 100 ng/mL SCF + 10 ng/mL IL-3 + 20 ng/mL IL-6 (all cytokines from Peprotech)

Maintenance media:

RPMI1640 + 10% FBS +50 µM 2-mercaptoethanol + cytokine (30 ng/mL GM-CSF)

Method:

1. CO_2 euthanize one mouse and collect the leg bones: femur, tibia and fibula. Remove as much of the tissue as possible and rinse in PBS.
2. Crush bones in PBS/0.5%BSA+2% mouse serum 2x(1 wet crush + 1 dry crush), collect into 50 mL Falcon tube
3. Triturate cell clumps by pipetting up and down with 5 mL tissue culture pipet
4. Filter cells/bone fragments through 40 µm strainer into another 50 ml Falcon tube
5. Spin 5 min at 1500 rpm
6. Resuspend in 4 mL PBS/0.5%BSA
7. Load on 3 mL Ficoll-Paque gradient
8. Spin 10 min at 2000 rpm
9. Collect all cells except bottom pellet
10. Dilute in 43 mL PBS/0.5%BSA
11. Spin 5 min at 2000 rpm
12. Resuspend in 3 mL PBS/0.5%BSA
13. Count cells
14. Spin 5 min at 1500 rpm
15. Resuspend at 40 µl/10^7 cells in PBS/0.5%BSA
16. Add 10 µl of biotinylated antibody cocktail/ 10^7 cells (Miltenyi Lineage Depletion Kit)
17. Mix, incubate 20 min at 4°C in coldroom (mix gently second time at 10 min)
18. Add 30µl of PBS/0.5%BSA per 10^7 cells
19. Add 20µl of magnetic beads/ 10^7 cells (Miltenyi Lineage Depletion Kit)
20. Mix, incubate 15min at 4°C (mix gently second time at 7 min)
21. Add 1 mL PBS/0.5%BSA
22. Spin 10 min at 300g

23. Equilibrate Miltenyi MS column on magnet with 500 µl PBS/0.5%BSA while cells are spinning
24. Resuspend cells in 500 µl PBS/0.5%BSA per 10^8 cells
25. Apply cells to column
26. Collect flowthrough – **this contains the lin$^-$ cells** (approximately $1x10^5$-$5x10^5$ cells depending on strain and age)
27. Wash column 2x with 750 µL PBS/0.5%BSA
28. Collect and pool washes with flowthru (total 2 mL)
29. Count cells
30. Spin 5 min at 1500rpm
31. Resuspend at 10^6 cells/ml in *progenitor outgrowth* media in 48 or 24 well (will grow/differentiate faster when more dense)
32. Incubate cells 24-48 h at 37°C
33. Count cells
34. Infect 2 x 10^5 cells/ml by spinoculation on retronectin coated 48 well plate in 0.3 mL at 3000rpm for 90min (*maintenance* media + conc. virus + 0.1% Lipofectamine2000)
35. Add 0.3 mL media after spin
36. Incubate overnight at 32°C in tissue culture incubator
37. Change ½ media next day
38. Passage non-adherent cells to new well with new media every 1-3 days

2.3 Lentivirus and Retrovirus Production

Preparation:

1. Culture HEK293T cells for at least 2 passages (~5 days) prior to transfection
3. Note: HEK293T cells have very poor adherency, especially during the virus production phase. Take great care in gently changing media, and use 1% gelatin treated flasks.

Method:

1. One day prior to transfection, passage HEK293T into a T75 treated with 1% gelatin solution (apply solution for ~10 mins, wash 2x with PBS) such that it will be 70-80% confluency on the day of transfection.
2. Prepare lipofectamine and DNA solution:
    a. 1.875 mL Opti-Mem + 75 µLLipofectamine 2000 (Invitrogen)
    b. 1.875 mL Opti-Mem + 30 µg DNA (Lentirivrus: 14 µg pLVX-insert, 6 µg VSV, 10 µg dR8.2; retrovirus: 15 µg pCL-Eco, 15 µg pMSCV-insert)
3. Allow solutions to incubate individually for 5 mins, then mix together, incubating the mixed solution for 20 mins.
4. Remove media from flask and add lipo/DNA solution (dilute to 10 mL Opti-MEM)
5. After 6 h, replace with fresh DMEM media.
6. Collect and change media every 24 h, up to 4 days, storing at 4°C.
    a. Check after 24 h for fluorescence of HEK293T cells if insertion construct has a fluorescent marker
    b. Be very careful, as the culture gets older, the cells become less adherent. Pipette gently and handle the flask with care to prevent the cells from sloughing off the surface

    c. *All materials from this point on should be treated with bleach solution*
7. Filter the collected media through a 0.45 µm filter.
8. Add Lenti-X-Concentrator or Retro-X-Concentrator (Clontech) (~13 mL to 40 mL of media), and incubate overnight at 4C
9. Spin at max speed (1500 g) for 45 min at 4C, and resuspend pellet in 400 µL of PBS.
10. Aliquot into 50 or 100 µL vials and freeze at -80°C until ready for use.

2.4 <u>Lentivirus and Retrovirus Transduction</u>

1. Coat infection wells (48 well plate) day before with 5-10 ug/mL retronectin (Clontech) in PBS overnight at 4°C.
2. Next day rinse coated wells 1x with PBS
3. Block non-specific binding with PBS+0.5% BSA for 30 min at room temperature.
4. Rinse blocked wells 2x with cell media
5. Mix in coated wells 20,000 cells in 200 µL growth media + 50 µL virus + 0.1% Lipofectamine 2000
6. Spin plate for 90 min at 1500 g at 30°C.
7. After spin add 300 µL growth media
8. Place cells overnight to 32°C cell culture incubator
9. Transfer next day to 37°C
10. Expand and assay for integration 5 days after infection. Split before if too dense after 3-4 days.

2.5 <u>qPCR</u>

*Note that all pipette tips used in these protocols should be filter tipped to prevent cross contamination

Reverse transcription to generate first-strand cDNA

1. Mix the following components:
    a. 4 µL Superscript VILO Mastermix (Invitrogen)
    b. 500 ng RNA
    c. X µL DEPC-treated water to 20 µL final volume
2. In a thermocycler, using the following program:
    a. 25°C for 10 min
    b. 42°C for 60 min
    c. 85°C for 5 min
3. Dilute the 20 µL solution into 380 µL water and store at -20°C until ready for use

Quantitative PCR (qPCR)

1. For each single run, prepare the following mixture. A mastermix can be made without the cDNA and pipetted into the wells of the plate (96 well thin walled hard shell PCR plates HSP9655 (Bio-Rad)). cDNA should be added individually to each well (scale as appropriate):
    a. 10 µL SsoFast Evogreen MM (Bio-Rad)
    b. 1 µL cDNA
    c. 0.8 µL Forward Primer (10 µM)
    d. 0.8 µL Reverse Primer (10 µM)
    e. 7.4 µL Water

2. Prepare triplicates for each gene per each cDNA sample.
3. Seal the plate with B seals (MSB1001, Bio-Rad)
4. In a Bio-Rad CFX96 thermocycler, set the following program:

Cycling Step	Temperature (°C)	Time (s)	# of Cycles
Enzyme Activation	95	30	1
Denaturation	95	15	40
Annealing	59	20	
Extension	72	40	
Melt Curve	65-95 (0.5 inc)	5/step	1

5. For each sample, calculate the ΔC_t values between actin and the gene of interest. To determine fold change from an untreated sample, use the following equation:

$$Fold\ Change = 2^{-(\Delta C_{t-sample} - \Delta C_{t-control})}$$

qPCR Primers

Gene	Sequence	PrimerBank ID*
Actin F	GGCTGTATTCCCCTCCATCG	6671509a1
Actin R	CCAGTTGGTAACAATGCCATGT	
Elane F	AGCAGTCCATTGTGTGAACGG	7657060a1
Elane R	CACAGCCTCCTCGGATGAAG	
Prtn3 F	ATGGCTGGAAGCTACCCATC	31981542a1
Prtn3 R	TGCCCACCTACAATCTTGGAG	
Ms4a3 F	GTGGTTCTGTTTATCAGCCCTT	18875420a1
Ms4a3 R	ACAGTGGGTAGCCTGTGTAGA	
Plac8 F	GCTCAGGCACCAACAGTTATC	21105853a1
Plac8 R	GCTGCCACTTGACATCCAAGA	
Emr1 (F4/80) F	TGACTCACCTTGTGGTCCTAA	2078508a1
Emr1 (F4/80) R	CTTCCCAGAATCCAGTCTTTCC	
IL12b F	TGGTTTGCCATCGTTTTGCTG	6680397a1
IL12b R	ACAGGTGAGGTTCACTGTTTCT	
iNOS (Nos 2) F	GTTCTCAGCCCAACAATACAAGA	6754872a1
iNOS R	GTGGACGGGTCGATGTCAC	
TNF F	CCCTCACACTCAGATCATCTTCT	7305585a1
TNF R	GCTACGACGTGGGCTACAG	
Arg1 F	CTCCAAGCCAAAGTCCTTAGAG	7106255a1
Arg1 R	AGGAGCTGTCATTAGGGACATC	
CD206 (Mrc1) F	CTCTGTTCAGCTATTGGACGC	6678932a1
CD206 R	CGGAATTTCTGGGATTCAGCTTC	
CCL17 F	TACCATGAGGTCACTTCAGATGC	225735578c1
CCL17 R	GCACTCTCGGCCTACATTGG	
*Each pair is identified with one PrimerBankID, taken from PrimerBank https://pga.mgh.harvard.edu/primerbank/)		

2.6 Flow Cytometry

Flow cytometry was conducted at the UCSF Flow Cytometry core on a BD Fortessa instrument. Cells were labelled with antibodies according to manufacturer instructions and the data was analyzed using FlowJo (FlowJo, LLC.). Cell sorting was conducted on a BD FACSAria instrument.

Cell Surface Labeling

1. Spin down 500,000+ cells, 400 g 4 min
2. Resuspend cells in PBS+0.5% FBS
3. Spin cells down again
4. Resuspend cells in PBS+0.5% FBS at 10^6 cells per 100µL
5. Mix 100 µL of cells with 1 µg of unlabeled anti-CD16/32 (FcBlock)
6. Incubate RT, 10 min
7. Add labeling antibody (0.1-1µg) (directly conjugated or biotinylated). For F4/80, use rat IgG2b κ isotype, anti-mouse F4/80 (0.2 µg per 10^6 cells in 100 µL), labelled with APC (Biolegend). Isotype used for control was unlabeled rat IgG2b κ isotype (Biolegend)
8. Incubate on ice 30-60 min
9. Add 200 ng labeled streptavidin if labeling antibody was biotinylated for 10min
10. Add 900 µL PBS+0.5% FBS
11. Spin down cells, 400 g 4 min
12. Resuspend labeled cells in 500 µL PBS+0.5% FBS
13. Read out on FACS (BD Fortessa at Parnassus Flow Cytometry Core, UCSF)

Have neg controls (no Ab, isotype specific non-specific Ab, no-expression-of-target cells)

Fluorescent Protein Cell Analysis

For cells that are fluorescently labeled, cells can be centrifuged and resuspended in D-PBS and analyzed on FACS without any further treatment.

DRAQ7 Staining for Dead Cells

Staining for live/dead cells was done using DRAQ7 (Abcam), which labels dead and apoptotic cells for flow cytometry, using the manufacturer protocols.
