# Supplementary material for: Clodronate Improves Survival of Transplanted Hoxb8 Myeloid Progenitors with Constitutively Active GMCSFR in Immunocompetent Mice
Source: Mol Ther Methods Clin Dev. 2017 Sep 7;7:60–73. doi: 10.1016/j.omtm.2017.08.007 (PMC5633862; doi:10.1016/j.omtm.2017.08.007)
Supplement: Document S2. Article plus Supplemental Information [file mmc2.pdf]

Molecular Therapy
Methods & Clinical Development
Original Article

Clodronate Improves Survival of Transplanted Hoxb8 Myeloid Progenitors with Constitutively Active GMCSFR in Immunocompetent Mice

Simon Lee,[1] Saul Kivimäe,[2] and Francis C. Szoka[1,2,3]

[1]UC Berkeley-UCSF Graduate Program in Bioengineering, University of California, San Francisco, San Francisco, CA 94143, USA; [2]Department of Bioengineering and Therapeutic Sciences, University of California, San Francisco, San Francisco, CA 94143, USA; [3]Department of Pharmaceutical Chemistry, University of California, San Francisco, San Francisco, CA 94143, USA

New methods to produce large numbers of myeloid progenitor cells, precursors to macrophages (MΦs), by maintaining Hoxb8 transcription factor activity[1] has reinvigorated interest in MΦ cell therapies. We generated Hoxb8-dependent myeloid progenitors (HDPs) by transducing lineage-negative bone marrow cells with a constitutively expressed Hoxb8 flanked by loxP. HDPs proliferate indefinitely and differentiate into MΦ when Hoxb8 is removed by a tamoxifen-inducible Cre. We genetically modified HDPs with a constitutively active GMCSF receptor and the tamoxifen-induced transcription factor IRF8, which we have termed "HDP-on." The HDP-on proliferates without GMCSF and differentiates into the MΦ upon exposure to tamoxifen and ruxolitinib (GMCSF inhibitor via JAK1/2 blockade). We quantified the biodistribution of HDPs transplanted via intraperitoneal injection into immunodeficient NCG mice with a luciferase reporter; HDPs are detected for 14 days in the peritoneal cavity, liver, spleen, kidney, bone marrow, brain, lung, heart, and blood. In immunocompetent BALB/c mice, HDP-on cells, but not HDPs, are detected 1 day post-transplantation in the peritoneal cavity. Pretreatment of BALB/c mice with liposomal clodronate significantly enhances survival at day 7 for HDPs and HDP-on cells in the peritoneal cavity, spleen, and liver, but cells are undetectable at day 14. Short-term post-transplantation survival of HDPs is significantly improved using HDP-on and liposomal clodronate, opening a path for MΦ-based therapeutics.

INTRODUCTION

Macrophages (MΦs) straddle the innate and adaptive immune systems, playing important roles in homeostatic tissue maintenance and in the immunopathology of many diseases, including cancer, bacterial infections, trauma, and arthritis.[2–5] To mediate these functions, MΦs are highly plastic, responding to changes in the environment by taking on inflammatory (M1) or regenerative (M2) phenotypes.[6–8] Furthermore, specialized MΦs perform critical functions in virtually all tissues, ranging from recycling heme in the kidney[9] to maintaining proper neuronal development in the brain.[10] Genetically engineering MΦs can augment their homeostatic roles, phenotypic plasticity, and diverse tissue niches to resolve dysregulated tissue functions. These applications may utilize gene therapy to produce therapeutic proteins[11–15] or deliver poorly soluble drugs.[16–18] As MΦ biology is more extensively explicated, the broad range of behaviors that MΦs possess provide an opportunity for engineering novel MΦ-based therapies.

The history of MΦ-based therapies began over 40 years ago,[19] but progress to date has been limited, due to the difficulty to generate the 10^7–10^8 MΦs required for human studies.[19] This barrier is partially because primary MΦs do not usually proliferate in vitro, unlike T cells and mesenchymal stem cells (MSCs), which facilely proliferate in vitro.[20–22] The earliest clinical trials for MΦ-based cell therapies for cancer treatment occurred over 30 years ago, where large numbers of autologous MΦs (10^8–10^9) were collected from patient blood, conditioned with granulocyte macrophage colony-stimulating factor (GMCSF) and interferon (IFN)γ in vitro, and reinfused.[19,23,24] At best, two or three doses of MΦ could be collected and infused. While there were minimal side effects, the efficacy was modest and mixed (for a review of MΦ-based cell therapies, see Lee et al.[19]). In more recent reports, tumor-derived MΦ-cell lines such as RAW264 or RAW309 have been used in animal studies.[11,25] These lines are problematic for therapeutic development, due to their tumorigenicity. Studies that use bone-marrow-derived MΦs lack a practical means to generate the MΦ numbers required for a therapy.[19]

There have been a few reports of altering the expression of transcription factors to induce self-renewal in MΦs and in myeloid progenitors, which can differentiate into MΦs. These include MafB and c-Maf double-knockout (Maf-DKO) MΦs[26] and Hoxb8-dependent myeloid progenitors (HDPs), which are held in a self-renewing state.[1,27] Maf-DKO MΦs and MΦs derived from HDPs are

Received 28 June 2017; accepted 31 August 2017;
http://dx.doi.org/10.1016/j.omtm.2017.08.007.

Correspondence: Francis C. Szoka, PhD, Department of Bioengineering and Therapeutic Sciences, University of California, San Francisco, 513 Parnassus Avenue, San Francisco, CA 94143-0446, USA.
E-mail: frank.szoka@ucsf.edu

This is an open access article under the CC BY license (http://creativecommons.org/licenses/by/4.0/).

functionally similar to endogenous MΦs from other sources[26,28] and are a good starting point for the development of MΦ-based therapeutics.

To perform further studies that required large numbers of MΦs, we were interested in simplifying and accelerating the process of generating large numbers of MΦs. The self-renewal capabilities of HDPs described by Wang and collaborators[1] were dependent upon a tamoxifen-dependent Hoxb8-ERT (estrogen receptor) and required culturing in 1 μM 4-hydroxytamoxifen (4-OHT). Removal of 4-OHT would stop Hoxb8 activity, and the HDP would differentiate into a MΦ. We asked whether a modified HDP with constitutively expressed Hoxb8 flanked by loxP sites and a 4-OHT-induced Cre recombinase could produce a self-renewing HDP that could undergo 4-OHT-inducible MΦ differentiation. HDPs also require the GMCSF to survive and proliferate. GMCSF signals through GMCSFR, and a single point mutation of GMCSFR (L452E) renders GMCSFR constitutively active and ablates the requirement for external GMCSF in myeloid cells.[29] We reasoned that the addition of constitutively active GMCSFR to HDPs would remove the need for the GMCSF to survive and proliferate.

To increase the rate of differentiation, we identified IRF8 as a transcription factor that is upregulated during MΦ differentiation.[30] By expressing 4-OHT-induced IRF8-ERT, we hypothesized that we could bypass the time required for IRF8 to be endogenously expressed and increase the differentiation rate of the HDP. By using a combination of lentivirus and retrovirus, we generated a modified HDP with constitutively active Hoxb8 and GMCSFR, as well as 4-OHT-inducible Cre and IRF8. To initiate differentiation, we cultured these modified HDP 4-OHT with ruxolitinib, a JAK1/2 inhibitor, to reduce GMCSFR activity and 4-OHT to activate Cre and IRF8. We demonstrate in this report that this new form of HDP, known as HDP-on, maintains the same growth and differentiation characteristics of the HDP, with cytokine- and 4-OHT-free self-renewal and 4-OHT- and ruxolitinib-inducible rapid differentiation into a MΦ.

Maf-DKO MΦs are nontumorigenic when injected into an immunodeficient mouse.[26] However, it is unclear whether Maf-DKO MΦs survive and engraft in this model. To our knowledge, there have been no reports of the in vivo survival of HDPs and HDP-derived MΦs (HDP-MΦs). We sought to quantitatively determine the survival potential of HDP-on cells and HDP-on-derived MΦs (HDP-on-MΦs) in immunodeficient NCG (lacking B, T, and natural killer [NK] cells) and immunocompetent BALB/c mice.

In this article, we describe two advances for the development of MΦ-based therapies: (1) a modified ex vivo method using Hoxb8, constitutively active GMCSFR, inducible Cre, and IRF8 (HDP-on) to facilely and rapidly generate large numbers of functional MΦ and (2) quantitatively validating the ability of clodronate-loaded liposome pretreatment to improve acute post-transplantation survival of HDP in immunocompetent BALB/c mice.

RESULTS

Hoxb8: A Method for Unlimited Myeloid Progenitors and MΦs

Primary MΦs are terminally differentiated cells and cannot be expanded in vitro. Recent reports described methods to circumvent this developmental block. One method, as described by Wang et al.[1] used tamoxifen-induced Hoxb8-ERT nuclear localization to hold a myeloid progenitor cell in a self-renewing state. This cell would differentiate into a MΦ when Hoxb8-ERT nuclear activity is reduced by the removal of 4-OHT from the media.[1] We modified this method by using a lentivirus to transduce primary lineage-negative (lin^-) bone marrow cells with a construct containing a constitutively expressed Hoxb8 flanked by loxP sites. Serial transduction with a Cre recombinase fused with ERT (Cre-ERT) endowed the cell with inducible Cre activity that could excise the Hoxb8 cassette with the addition of 4-OHT. This enables the differentiation of the progenitor cell toward a MΦ state that can be specified by the presence of GMCSF in the growth media (Figure 1). The construct containing Cre-ERT also contained luciferase, a traceable marker, to allow the detection of live HDPs. Cells that die do not contribute to luciferase activity, as the enzyme has poor serum stability and has a very short circulatory half-life of under 20 min.[33] This reporter enables biodistribution studies by allowing for the total number of live cells to be determined from organ lysates. This cell, the HDP, formed the basis of our studies. HDPs require the GMCSF to proliferate and survive, which was provided by using a GMCSF supplement generated from the conditioned media of GMCSF-expressing L929 cells. Furthermore, HDPs can be cultured indefinitely in suspension culture to high densities (1–2 $\times$ 10^6/mL) and then differentiated into adherent MΦs by adding 4-OHT to the media for 10 days. This model allows for the generation of the high number of HDPs and HDP-MΦs required for the development of a MΦ-based therapy.

Constitutively Active GMCSFR HDPs Differentiate into MΦs

To further enhance the performance of HDPs, two additional genetic modifications were made: a constitutively active GMCSFR and IRF8-ERT, forming the HDP-on (Figure 1). Constitutively active GMCSFR results from a single point mutation (L452E)[29] and, in myeloid cells and MΦs, results in survival and proliferation without the need for GMCSF. We hypothesize that this modification could potentially increase the overall in vivo survival, potential due to a limited pool of GMCSF in vivo.[34] Additionally, reducing the need for cytokine (either recombinant or from conditioned media) reduces the cost of materials to maintain these cells. HDP-on cells in media without GMCSF supplement proliferate as rapidly as HDPs with GMCSF supplement, with a doubling rate of ∼12 hr (Figure 2A). This proliferation rate enables the rapid generation of a high number of cells for differentiation into MΦs. To further enhance the ability to rapidly generate MΦs, IRF8-ERT was also added to HDPs. IRF8 is a transcription factor that is upregulated during MΦ differentiation,[30] and constitutive expression of 4-OHT-inducible IRF8 results in a faster differentiation process by reducing the time for endogenous IRF8 to be expressed and migrate to the nucleus. In comparison to HDPs, which lack IRF8-ERT, the addition of IRF8-ERT reduces the time required for

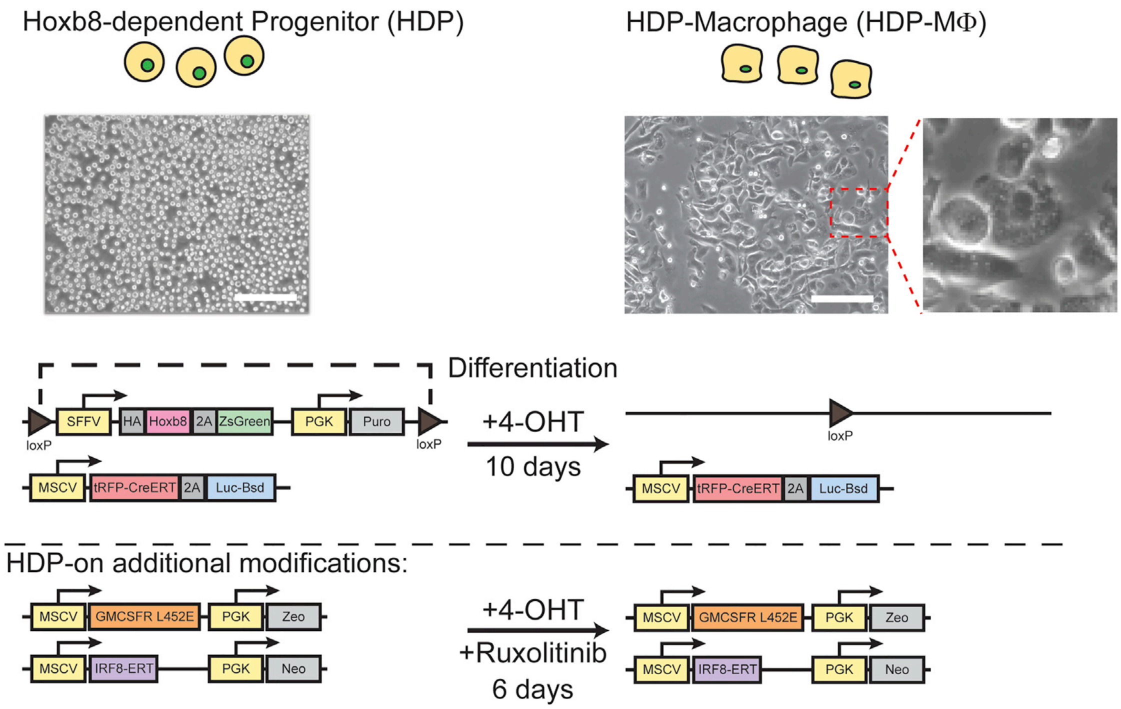

Figure 1. Generation of HDPs and HDP-MΦs

Lin$^-$ cells were isolated from bone marrow and transduced using a lentivirus with a Hoxb8 construct flanked by loxP sites. Subsequent transduction with retrovirus inserted a Cre-ERT recombinase with a luciferase reporter, which, respectively, served to induce the excision of Hoxb8 in the presence of 4-hydroxytamoxifen (4-OHT) and provided a reporter to be quantified for biodistribution studies. Excision of Hoxb8 led to differentiation of HDPs into HDP-MΦs in 10 days. Modified HDPs, known as HDP-on cells, were serially transduced with retroviruses encoding a constitutively active GMCSFR and IRF8-ERT. HDP-on cells differentiate into HDP-on-MΦs in 6 days when treated with 4-OHT and ruxolitinib. Scale bar, 150 μm.

differentiation from 10 to 6 days, further simplifying the amount of processing required to generate MΦs. While HDPs require only 4-OHT to differentiate, HDP-on cells also require ruxolitinib, a Jak2 inhibitor that inhibits GMCSFR activity. Gene expression analysis of HDP-on cells differentiated for 6 days in 40 nM 4-OHT and 1 μM ruxolitinib by qPCR for MΦ- and HDP-specific genes[1,27] reveals upregulation of the MΦ marker F4/80 (*Emr1*) and significant downregulation of HDP genes *Elane*, *Prtn3*, *Ms4a3*, and *Plac8* (Figure 2B). Flow-cytometric analysis of differentiating HDP-on cells over 6 days also demonstrates increased surface F4/80 expression (Figure 2C), a hallmark of MΦ differentiation. Ruxolitinib alone does not affect F4/80 expression, while 4-OHT alone increases F4/80 expression to a smaller degree. Combination treatment results in significantly more F4/80 expression by day 6, demonstrating the synergistic effects of ruxolitinib and 4-OHT on rapidly differentiating HDP-on cells into MΦs. Based upon the gene expression and flow cytometry of differentiated HDP-on cells, we believe that HDP-on cells efficiently differentiate into MΦs and could serve as a model for generating MΦs for further studies.

HDP-on-MΦs Retain M1/M2 Polarization Responses and Remain Highly Phagocytic

We determined whether HDP-on-MΦs retained typical MΦ behaviors and phenotypes, using functional assays of phenotypic polarization and phagocytosis. One of the greatest potentials for MΦ-based therapies is to harness the plasticity of MΦs by polarization toward inflammatory (M1) or regenerative (M2) phenotypes. The broad spectrum of phenotypes demonstrates the potential applications of MΦs for a wide variety of conditions. It is important to note that the M1/M2 paradigm is not necessarily a binary distinction[6,35] but, rather, describes a continuum of behaviors. However, there exist commonly accepted standards to describe M1-like and M2-like behaviors that can be elicited using specific polarization inducers. These methods were used to polarize and characterize HDP-on-MΦs. HDP-on-MΦs treated with lipopolysaccharide (LPS), an M1 inducer, responded by upregulating known M1-associated genes *IL12b*, *iNOS*, and *TNF* (Figure 3A). Similarly, treatment with interleukin-4 (IL-4), an M2 inducer, upregulated the M2-associated genes *Arg1*, *CD206*, and *CCL17* (Figure 3A). This was not observed in undifferentiated HDP-on cells treated with LPS or IL-4 (Figure S1). Treatment of M1/M2-polarized HDP-on-MΦs with the opposing polarization inducer also resulted in polarization to the opposing phenotype (Figure S2). Based on these results, it is clear that HDP-on-MΦs retain the plasticity that exists in conventionally derived MΦs.

Another key function of MΦs is their ability to phagocytose other cells or materials. MΦs have also been proposed to act as drug carriers,[18,36,37] so demonstrating this ability is key to enabling this

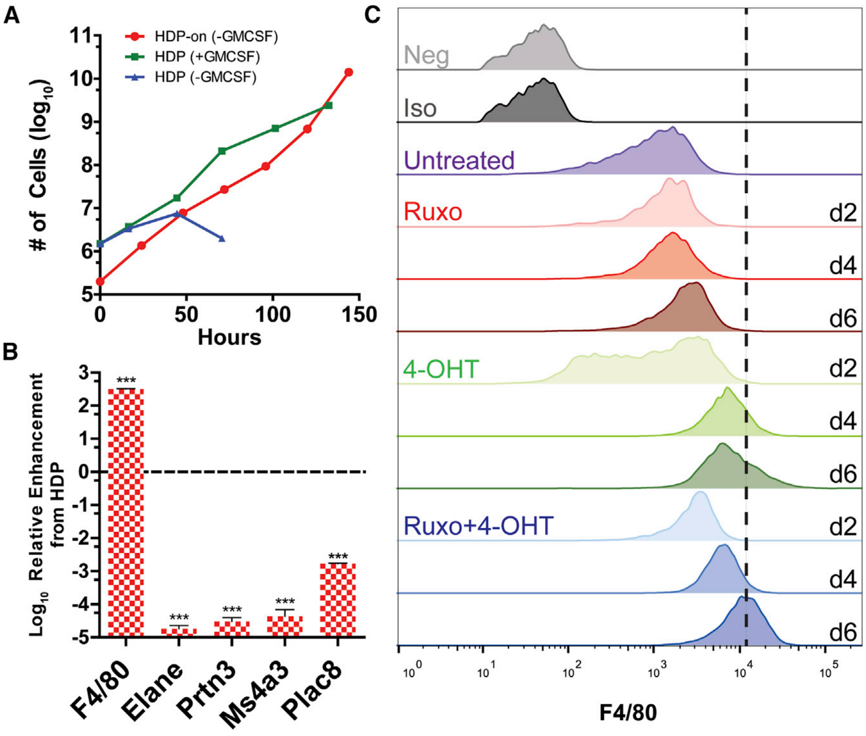

Figure 2. Characterization of HDP-on Cells and HDP-on-MΦs

(A) Proliferation curves of HDP-on cells and HDPs with and without GMCSF supplement. (B) Gene expression analysis by qPCR of known MΦ and HDP genes confirm the MΦ status of HDP-on cells treated with 40 nM 4-OHT and 1 μM ruxolitinib for 6 days. Expression is presented as fold enhancement from untreated HDP-on cells (n = 3). ***$p < 0.001$. (C) F4/80 surface expression by flow cytometry. HDP-on cells were cultured with or without 40 nM 4-OHT or 1 μM ruxolitinib (Ruxo) and assayed for F4/80 expression at 2, 4, and 6 days. Neg, unlabeled cells; Iso, cells labeled with APC-labeled isotype control.

form of MΦ cell therapy. HDP-on-MΦs were co-incubated with fluorescent DiD-labeled liposomes for 3 hr in serum-free media, and total lipid uptake was determined by measuring the total DiD fluorescence and comparing to a standard curve. RAW264, a MΦ cell line, was used as a positive control. Quantitative uptake studies demonstrated that HDP-on-MΦ and RAW264 cells exhibited a similar ability to phagocytose negatively charged liposomes (Figure 3B). Fluorescent imaging of HDP-MΦs incubated with fluorescent HPTS liposomes and TagBFP-expressing *E. coli* also shows that HDP-on-MΦs are highly phagocytic for both liposomes and *E. coli* (Figure 3C).

Undifferentiated HDP-on Cells and HDP-on-MΦs Survive at Least 7 Days in Immunodeficient Mice

We next determined the survival potential of HDP-on and HDP-on-MΦs injected into the immunodeficient NCG strain of mice that lack B, T, and NK cells to determine whether HDP-MΦs and HDP-on-MΦs survive in mice lacking an immune system. In some animals, live cells were detected in the peritoneal cavity and spleen (Figure 4). In the peritoneal cavity, undifferentiated HDP-on cells demonstrated significantly greater survival than HDPs (151% ± 4% versus 107% ± 4% of the injected cells, or 7.55×10^6 cells versus 5.35×10^6 cells; $p < 0.01$). However, this trend was reversed in the spleen, where there were significantly fewer HDP-on cells than HDPs (11% versus 2% of the injected cells, or 5×10^5 cells versus 1×10^5 cells; $p < 0.01$).

MΦs derived from either HDPs or HDP-on cells had significantly reduced survival in the peritoneal cavity (4.4% ± 0.3%, 2.2×10^5 cells; and 19.4% ± 3.3%, 9.7×10^5 cells, respectively) when compared to the respective parental cell. Neither MΦ type was detected in the spleen. Other tissues were analyzed, including the liver, heart, bone marrow, lungs, brain, kidney, and blood, but the combined percentages of injected cells detected across these tissues was below 1.5% of injected cells in all conditions tested (Figure S3).

Furthermore, there was a differential in the number of cells recovered between the peritoneal cavity and spleen, with HDP-on cells having a greater number of cells in the peritoneal cavity than HDPs. The opposite was true in the spleen (Figure 4). Overall, these experiments indicate that both HDPs and HDP-MΦs with or without HDP-on modifications can survive at least 7 days in immunodeficient animals.

Clodronate Pretreatment Improves Survival of HDP-on Cells in Immunocompetent Mice

Due to the significantly higher survival of HDPs and HDP-on cells when compared to the differentiated MΦs in immunodeficient NCG mice, we focused on determining the survival of undifferentiated HDPs and HDP-on cells in healthy immunocompetent BALB/c mice (Figure 5). HDPs were not detected in any tissues at 1 or 7 days post-transplantation. HDP-on cells had significantly higher survival in the peritoneal cavity 1 day post-transplantation, compared to HDPs (107% ± 21% or 5.35×10^6 cells; $p < 0.001$). However, survival of HDP-on cells in other tissues was limited: at 1 day post-transplantation, <1% was detected in the spleen or liver; and at 7 days post-transplantation, <0.5% was detected in the liver, and no cells were detected in the peritoneal cavity or spleen. For other tissues, including brain, lung, heart, bone marrow, liver, spleen, kidneys, and blood, the total combined percentage of injected cells detected for either HDPs or HDP-on cells was below 1% of the injected cells (Figures S4 and S5).

We sought to improve the survival of either HDPs or HDP-on cells and hypothesized that the removal of endogenous MΦs may improve survival, since improved survival of other cell types has been reported when animals are pretreated with MΦ-killing liposomal clodronate.[38]

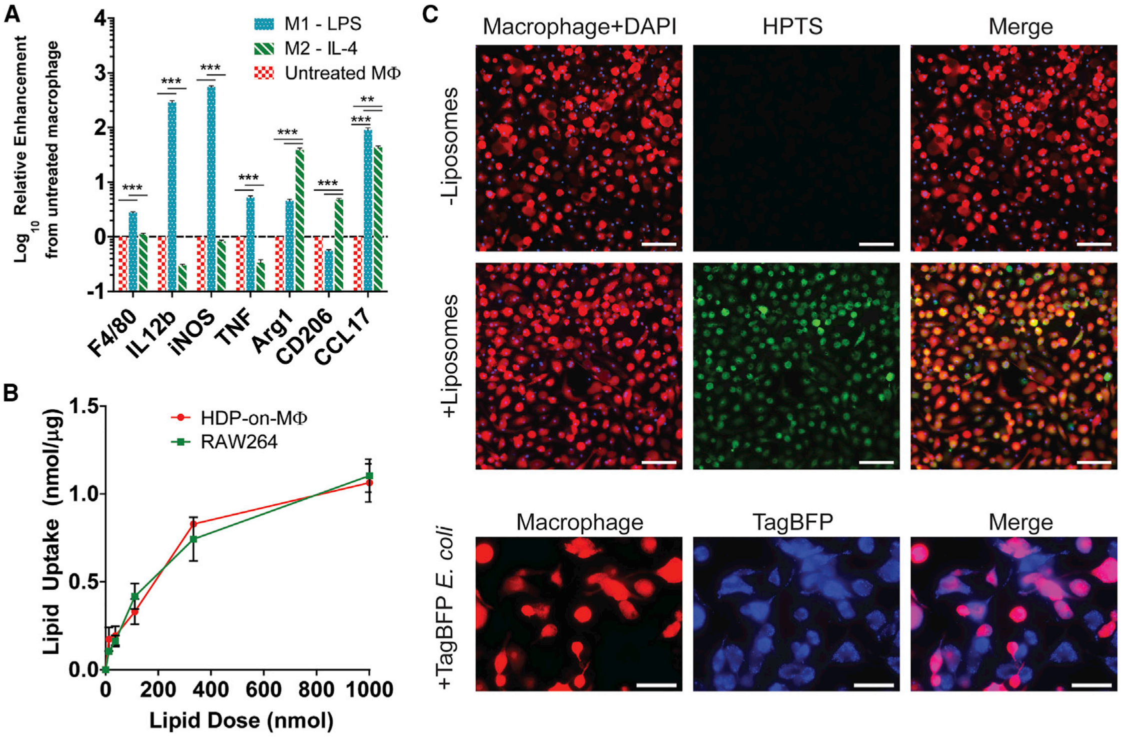

Figure 3. Functional Analysis of HDP-on-MΦs for MΦ Behaviors

(A) HDP-on-MΦs respond to canonical M1 and M2 inducers, LPS and IL-4. MΦs were treated overnight with 10 ng/mL LPS or 100 ng/mL IL-4, and gene expression of established M1 and M2 genes (M1: *IL12b*, *iNOS*, and *TNF*; M2: *Arg1*, *CD206*, and *CCL17*) was measured by qPCR. Fold enhancement is expressed relative to untreated HDP-on-MΦs (n = 3). $^{**}p < 0.01$; $^{***}p < 0.001$. (B) HDP-on-MΦs phagocytose DiD-labeled liposomes at a similar rate as the MΦ cell line, RAW264. Lipid uptake was normalized by total protein from lysed cells (N = 3 per condition). (C) Fluorescent images of HDP-derived MΦs incubated with HPTS-fluorescent liposomes and TagBFP-expressing *E. coli*. Scale bars, 150 μm (for liposome uptake) and 100 μm (for *E. coli*). Error bars are expressed as SE.

Endogenous MΦs are found in tissue niches that transplanted HDPs might be able to occupy. Successful transplantation of MΦs has been reported in *Csf2rb*-KO mice, which have impaired MΦ activity and reduced numbers of MΦs.[34,39] Additionally, F4/80$^+$ cells phagocytose stem-cell-derived hematopoietic progenitor cells[38] and could be involved in removing HDPs. Hence, the removal of endogenous MΦs may be beneficial for HDPs by creating a niche and interfering with active removal by endogenous MΦs. Endogenous MΦs can be transiently removed from the peritoneal cavity, liver, spleen, and blood with liposomal clodronate.[40,42,43] We accomplished this by injecting 100 μL liposomal clodronate (5 mg/mL) intraperitoneally (i.p.) 1 day and 4 days prior to the injection of cells.

At 1 day post-transplantation, liposomal clodronate pretreatment only increased survival in the peritoneal cavity for HDPs, and no benefit was detected in other tissues. Compared to HDP survival in untreated mice, liposomal clodronate pretreatment significantly improved the survival of HDPs in the peritoneal cavity (95% ± 7.1% or 4.75×10^6 cells versus undetectable; $p < 0.001$). This improvement was sustained at 7 days post-transplantation, with 85% ± 15% (4.25×10^6 cells versus undetectable; $p < 0.001$) of the injected cells detected in the peritoneal cavity. HDP-on cells did not experience any significant improvement in the peritoneal cavity with liposomal clodronate pretreatment at 1 day post-transplantation when compared to untreated animals (90% ± 10% or 4.5×10^6 cells versus 107% ± 20% or 5.35×10^6 cells). However, HDP-on cells experienced significant enhancement in survival at 7 days post-transplantation in the peritoneal cavity when compared to animals that did not undergo liposomal clodronate pretreatment (60% ± 17% or 3×10^6 cells versus undetectable; $p < 0.05$).

No improvements were seen in tissues other than the peritoneal cavity at 1 day post-transplantation. At 7 days post-transplantation, improvement in cell numbers was seen in the spleen and liver for both HDPs and HDP-on cells. In the spleen, HDPs increased from undetectable to 21.2% ± 2.7% (1.06×10^6 cells; $p < 0.001$), and HDP-on cells increased from undetectable to 3.4% ± 0.9% (1.5×10^5 cells; not significant). The enhancement was more modest in the liver, with HDPs increasing from undetectable to 1.35% ± 0.1% (6.75×10^4 cells; $p < 0.001$) and HDP-on cells increasing from 0.22% ± 0.08% (1.1×10^4 cells) to 0.86% ± 0.18% (4.3×10^4 cells; $p < 0.01$). In other tissues tested, there was no measurable benefit with respect to the cell number found in the tissue (Figures S4 and S5).

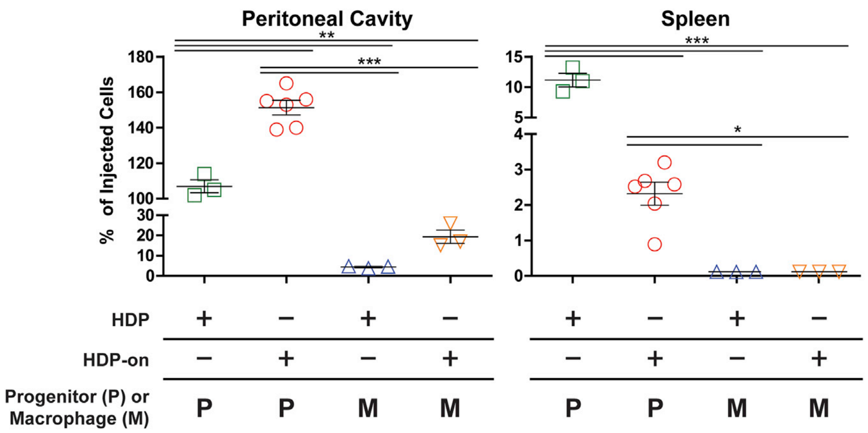

Figure 4. Biodistribution of HDPs, HDP-on Cells, HDP-MΦs, and HDP-on-MΦs in Immunodeficient NCG Mice 7 Days Post-injection

Peritoneal cavity and spleen are shown (see Figure S3 for other tissues). Mice were injected i.p. with 5 × 10^6 cells in 500 μL RPMI with a 28G syringe and euthanized after 7 days, and tissues were analyzed for luciferase activity. Error bars are expressed as SE. Statistics: N = 6 for HDP-on, and N = 3 for other conditions, one-way ANOVA with Bonferroni post-analysis. *p < 0.05; **p < 0.01; ***p < 0.001.

To summarize, in immunocompetent BALB/c mice, the HDP-on modifications significantly improved the number of HDPs that survived in the peritoneal cavity 1 day post-transplantation via the intraperitoneal route of administration. This benefit is not maintained, because no cells were detected at 7 days. Liposomal clodronate pretreatment to remove endogenous MΦs increased the peritoneal cavity survival at 1 day post-transplantation of HDPs to levels similar to those of HDP-on cells. Liposomal clodronate pretreatment improved the survival of HDPs and HDP-on cells at 7 days post-transplantation in the peritoneal cavity, spleen, and liver. MΦs differentiated from HDPs and HDP-on cells did not show increased survival in any tissue in the liposomal-clodronate-treated animals (Figure S6).

Liposomal Clodronate Pretreatment Does Not Enhance Post-transplantation Cell Survival in Immunocompetent Mice beyond 7 Days

We determined whether liposomal clodronate pretreatment enabled the long-term survival of HDP-on cells beyond 7 days. HDP-on cells were injected i.p. into NCG mice that were not treated with liposomal clodronate and in liposomal-clodronate-pretreated BALB/c mice. Liposomal clodronate was not used in NCG mice due to unacceptably high mortality, even at reduced doses of 15 mg/kg (data not shown). To determine the kinetics of in vivo survival post-transplantation for liposomal-clodronate-pretreated BALB/c mice, mice were euthanized at multiple time points and their data were combined with data from the previous section to determine the biodistribution at 1, 3, 7, and 14 days post-transplantation. Similarly, biodistribution was also performed in NCG mice at 7 and 14 days post-transplantation.

The long-term survival of HDP-on cells was limited in liposomal-clodronate-treated BALB/c mice (Figure 6). In these mice, the number of live cells detected in the peritoneal cavity decreased steadily 1 day post-transplantation and were undetectable at 14 days. In the liver, spleen, kidney, and bone marrow, the number of HDP-on cells increased from 1 day to 7 days post-transplantation. The total percentage of injected cells detected in these tissues at 7 days ranged from a low of 0.4% in the kidney to a high of 4% in the spleen. However, similar to the peritoneal cavity, 14 days post-transplantation, no cells were detected in these tissues. At no point were HDP-on cells detected in the brain, heart, lung, or blood in BALB/c mice (Figure S7).

In NCG mice, the number of live HDP-on cells increased across all tissues between 7 days and 14 days post-transplantation. In the peritoneal cavity, the total percentage of injected cells detected increased from 151% ± 4% (7.55 × 10^6 cells) at 7 days to 718% ± 208% (35.9 × 10^6 cells) at 14 days post-transplantation, representing a 4.75-fold enhancement. The relative increase between 7 and 14 days was also high in the other organs, with 75-, 4-, 26-, and 29-fold enhancement, in the liver, spleen, kidney, and bone marrow, respectively. HDP-on cells were detected at 14 days in other tissues (brain, blood, and lungs), which did not show live cells in any other conditions we have tested (Figure S8). Furthermore, at 14 days, NCG mice displayed significant morbidity and mortality and were euthanized in accordance with UCSF IACUC protocols.

DISCUSSION

In this study, we demonstrated that (1) HDPs modified with a constructively active GMCSFR and IRF8-ERT (HDP-on cells) can self-renew without cytokine and rapidly differentiate into MΦs; (2) HDP-on-MΦs retain MΦ-like behaviors, including phagocytosis and M1/M2 polarization; (3) HDP-on cells and HDP-on-MΦs persist in immunodeficient NCG mice for at least 7 days, using a quantitative luciferase-based assay; and (4) liposomal clodronate pretreatment enhances the survival of transplanted HDP-on cells in healthy BALB/c mice for at least 7 days.

We modified a method developed by Wang et al.[1,27] to hold myeloid progenitors in a self-renewal state by inserting a constitutively expressed Hoxb8 construct flanked by loxP sites. Removal of Hoxb8 was performed by a tamoxifen-induced Cre recombinase and induced differentiation of the progenitor into a MΦ. Addition of HDP-on modifications produced progenitors capable of self-renewal without GMCSF and enabled accelerated differentiation. F4/80 surface expression by flow cytometry demonstrated that HDP-on cells differentiate into MΦs in 6 days when treated with 40 nM 4-OHT and 1 μM

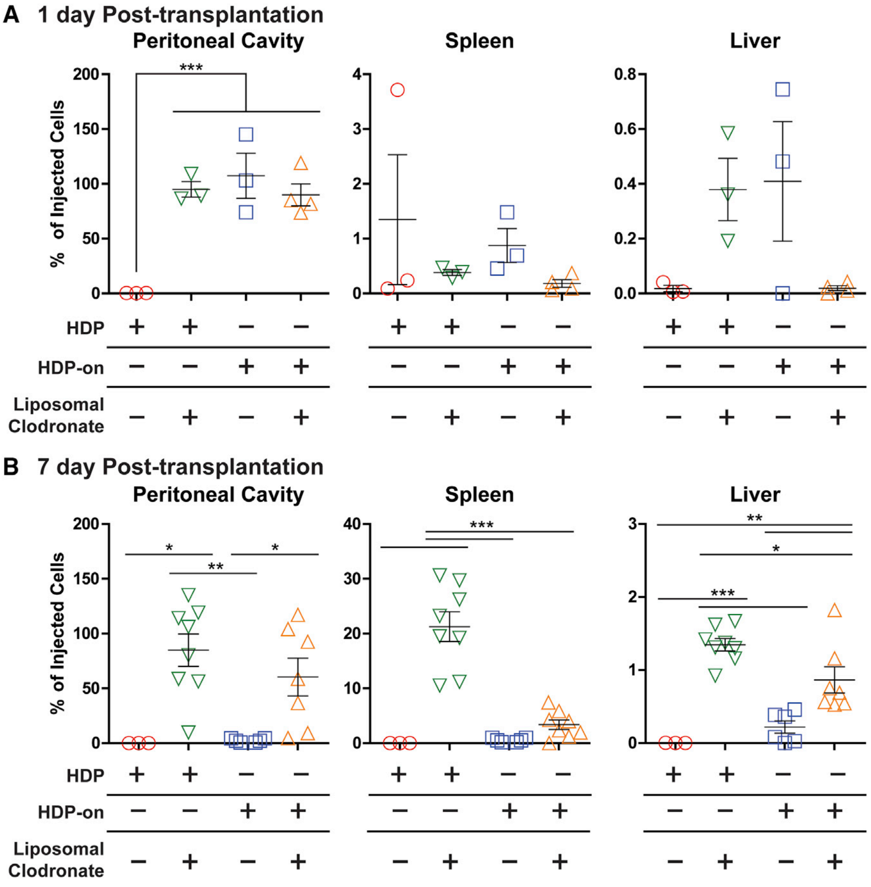

Figure 5. Biodistribution of Undifferentiated HDPs and HDP-on Cells in Mice Pretreated with Liposomal Clodronate

(A and B) Healthy BALB/c mice were injected i.p. with 5×10^6 cells in 500 μL RPMI with a 28G syringe and euthanized after (A) 1 day or (B) 7 days, and tissues were analyzed for luciferase activity. Statistics: N = 3 for all conditions at 1 day post-transplantation and HDPs with no liposomal clodronate at 7 days post-transplantation; N = 6 for all other 7-day time points; one-way ANOVA with Bonferroni post-analysis. Error bars are expressed as SE. *p < 0.05; **p < 0.01; ***p < 0.001. See also Figures S4 and S5. Error bars are expressed as SE.

ruxolitinib. These HDP-on-MΦs responded to M1/M2 polarization signals and were phagocytic for fluorescent liposomes.

Previous reports using MΦs for cell-based therapies[19] have used bone-marrow-derived MΦs[34,39] or immortalized RAW264 cell lines,[11,25] which are either limited in number or highly transformed. Using HDPs, we were able to easily genetically engineer HDP cells using retrovirus to tailor the cell for various applications, generate large numbers of HDP cells, and differentiate these cells into functional MΦs. The ability to generate large numbers of cells is especially important. Other cell-based therapies, including T cell and stem cell therapies, rely on self-renewing cell types to generate therapeutic doses of the cell for animal and clinical studies.[20–22] Primary MΦs do not normally proliferate in vitro, so the methods described here reduce barriers for the evaluation of MΦ cell-based therapies. Having these cells enabled us to study the biodistribution of HDPs and MΦs in immunocompromised NCG and healthy BALB/c mice.

There has been relative paucity in quantitative approaches to determine the biodistribution of MΦ-based therapies.[19] Most studies have relied on qualitative measures such as a biological functional response, histology, or flow cytometry. While these methods may determine the presence of the transplanted cell, a quantitative approach is needed to measure biodistribution, dose response, and cell survival to better assess and develop cell-based therapies. We used a luciferase-based system, as it provided a quantitative method to count only live cells due to poor serum stability of luciferase when a cell dies.

Endogenous MΦs may serve as barriers to the post-transplantation survival of HDP-on cells and HDP-on MΦs. This may be due to the highly phagocytic nature of MΦs, or the lack of a tissue niche for transplanted cells to survive. Phagocytic endogenous MΦs are responsible for the low post-transplantation survival of embryonic-stem-cell-derived hematopoietic progenitors in the highly immunodeficient NOD/SCID (non-obese diabetic/severe combined immunodeficiency) mouse model.[38] Long-term post-transplantation survival of wild-type bone-marrow-derived MΦs have been observed in a *Csf2rb* KO mouse, which possesses small numbers of dysfunctional MΦs.[34,39] In this model, the lack of functional MΦs may have allowed the functional MΦs to engraft and survive. Much like how immunoablation by chemoablation or sublethal radiation is used to prepare hosts for the transplantation of T cells or hematopoietic stem cells,[21] the removal of endogenous MΦs may generate tissue niches for transplanted HDP-on cells and HDP-on-MΦs to enable long-term survival. Administration of liposomal clodronate has been shown to temporarily eliminate endogenous MΦs,[40] and we observed that pretreating mice with liposomal clodronate increases the post-transplantation survival of HDP-on cells but not HDP-on-MΦs.

We conducted biodistribution studies by injecting HDPs, HDP-on cells, HDP-MΦs and HDP-on-MΦs i.p. in immunodeficient NCG and immunocompetent BALB/c mice. In NCG mice, HDPs survived significantly better than MΦs, with HDP-on cells having a higher number of injected cells than HDPs in the peritoneal cavity (120% versus 100%) 7 days post-transplantation. In BALB/c mice, HDPs were undetectable across all tissues 1 and 7 days post-transplantation. Addition of the HDP-on modifications enabled the detection of

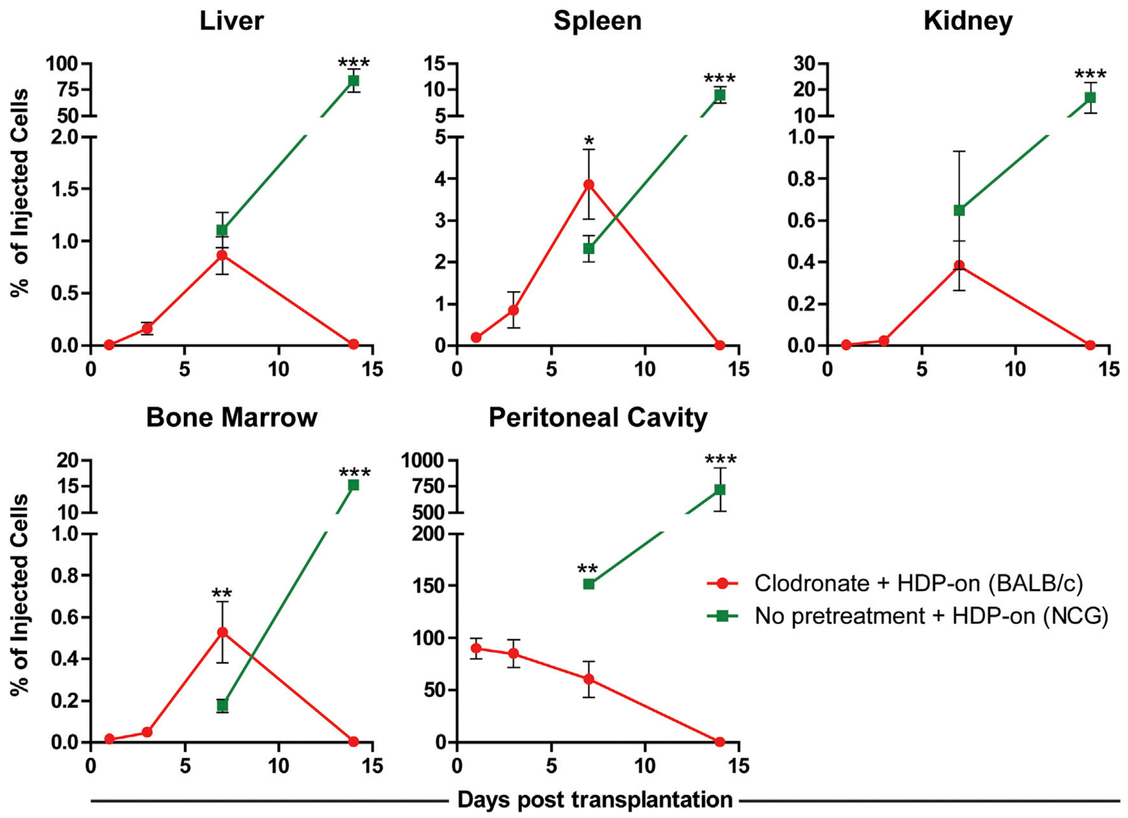

Figure 6. Biodistribution of HDP-on in NCG Mice and Liposomal-Clodronate-Treated BALB/c Mice

Biodistribution was determined by luciferase activity in tissue lysates up to 14 days post-injection. Mice were injected i.p. with 5×10^6 cells and euthanized at 1, 3, 7, or 14 days. Error bars are expressed as SE. N ≥ 3 for each condition; statistical comparisons were made within the same time points. *$p < 0.05$; **$p < 0.01$; ***$p < 0.001$.

HDPs in the peritoneal cavity up to 1 day post-transplantation. Pretreatment of animals with liposomal clodronate improved the survival of both HDPs and HDP-on cells; with cells detected in multiple tissues, including the peritoneal cavity, liver, and spleen up to 7 days post-transplantation. Substantial proliferation of HDP-on cells in NCG mice was detected across most tissues, while no cells were detected in the liposomal-clodronate-treated BALB/c mice 14 days post-transplantation.

The robust survival in NCG mice indicated that HDPs can survive with only endogenous cytokines in vivo. Detecting more than 100% of the injected cells is very significant, as it also indicates that cells are proliferating in vivo. The substantial reduction in surviving MΦs in both NCG and BALB/c mice may be indicative of the transplantation process being more deleterious on MΦs or that the peritoneal cavity may lack the survival factors required to support the survival of the 5×10^6 MΦs that were transplanted. Additionally, biodistribution studies conducted by transplanting cells via the intravenous route did not result in any cells detected beyond 1 day in any organ (data not shown).

Administration of liposomal clodronate via the peritoneal cavity temporarily removes MΦs from the peritoneal cavity, spleen, liver, and blood.[40] Thus, removal of endogenous MΦs may be responsible for the improved HDP survival in liposomal-clodronate-treated BALB/c mice in the peritoneal cavity (60%–85% of total injected cells at 7 days), liver (0.9%–1.3%), spleen (3%–21%), kidney (<1%), and bone marrow (<1%). In support of this observation, MΦs are primarily responsible for removing embryonic-stem-cell-derived hematopoietic progenitors.[38] The enhanced HDP survival after liposomal clodronate treatment is probably due to the removal of endogenous MΦs. The endogenous MΦs may phagocytose transplanted HDPs or occupy tissue niches required by HDPs for longer term survival.[8]

In BALB/c mice pretreated with liposomal clodronate, the number of HDP-on cells steadily increased in the spleen, liver, kidneys, and bone marrow site over 7 days, indicating migration from the peritoneal cavity to other tissues. However, no HDP-on cells were detected at 14 days in any tissues. We believe that the loss of HDP-on cells between 7 and 14 days is possibly related to the re-establishment of endogenous tissue MΦ populations. After liposomal clodronate treatment, MΦs repopulate in the mouse spleen and rat liver within 7–14 days.[44,45] The returning endogenous MΦs may phagocytose the transplanted HDP-on cells and/or reoccupy the niche. To improve long-term survival of HDP-on cells, repeated clodronate treatment may be required post-transplantation. Liposomal

clodronate pretreatment did not improve MΦ survival, though this may be due to residual liposomal clodronate killing transplanted MΦs. Liposomal clodronate injected intravenously (i.v.) is not detectable in the blood 3 hr post-injection,[46] but administration i.p. may extend the overall clearance time. This may be addressed by a different injection schedule to allow for the clearance of liposomal clodronate before transplantation of MΦs.

Long-term survival (at least 9 months) of transplanted bone-marrow-derived MΦ has been demonstrated in the lungs of *Csf2rb*$^{-/-}$ mice.[34,39] These mice have a significantly reduced number of endogenous lung MΦs, which could result in niches for the transplanted MΦs to occupy. Furthermore, *Csf2rb* encodes for the GMCSF receptor, which provides both survival and proliferation signals to MΦs in the presence of GMCSF. Thus, the wild-type MΦs had a survival and proliferative advantage over the endogenous *Csf2rb*$^{-/-}$ MΦs and, over time, were able to outcompete the endogenous MΦs for the remaining niches. The addition of HDP-on modifications to HDPs possibly provides a proliferative/survival advantage that did not appear to mimic the effect observed in these studies. However, our studies used a different route of administration in healthy mice. Long-term survival of transplanted MΦs may be more successful in appropriate disease models with impaired MΦs.

Based upon the survival of HDPs in NCG mice beyond 7 days, it is clear that the immunodeficiencies of NCG mice have an impact on the survival of HDPs. Immunodeficient NCG mice possess MΦs, yet HDP-on cells survive beyond 7 days. This would imply that B, T, or NK cell activity may also be responsible for the loss of transplanted HDPs in BALB/c mice. Syngeneic cells were injected into BALB/c mice, but the HDPs were extensively modified to express foreign proteins, including fluorescent proteins, antibiotic resistance markers, and luciferase. These types of foreign proteins can be immunogenic and lead to the rejection of transplanted cells.[47–49]

Rejection of GFP-expressing cells has been strongly associated with T cells,[47] and NK cells target stem-cell-derived hematopoietic progenitors.[50] Swijnenburg and colleagues performed studies with human embryonic stem cell xenografts into mice and dissected the immune response to identify CD4^{+} T cells and the adaptive immunity response as responsible for the loss of transplanted cells.[51,52] Using histology and flow cytometry on tissue digests, they identified significant immune cell (T, B, MΦ, and neutrophil) infiltration in the injection site.[52] Furthermore, when immunocompetent mice received a second injection of stem cells, no cells were detected at 3 days post-injection.[51] Similarly, in BALB/c mice that received an additional round of liposomal clodronate and a second HDP-on injection (liposomal clodronate on day −4 and day −1, HDP-on injection on day 0, second round of liposomal clodronate on days 7 and 11, and second HDP-on injection on day 12; Figure S9), no cells were detected in any tissue 3 days after the second dose. Additionally, in mice that received the xenograft, splenocytes secreted more IL-4 than IFNγ, which, respectively, corresponded to Th2 (humoral immunity) and Th1 (cellular immunity) responses. Immunoglobulin (Ig)M levels were also significantly higher after transplantation. Swijnenburg and colleagues performed xenograft survival studies in Nude, CD4^{-}, and CD8^{-} mice to determine CD4^{+} T cells as mediators for removing the xenograft.[51] Finally, they identified that a pretreatment strategy of a combination of tacrolimus (a calceinurin inhibitor) and rapamycin enabled the xenograft to survive for at least 28 days.[51]

We believe that the combination of humoral and cellular immune responses they observed may also be applicable to HDP-on cells in syngeneic animals. Further experiments, using an array of immunodeficient mice or B/T/NK-cell-depletion strategies, are required to isolate the cell type(s) responsible for the rejection of HDP-on cells. A similar drug treatment approach, targeting T cell activity, may also enhance the survival of HDPs.[53] Other methods, such as blockade of co-stimulatory molecules (anti-LFA1, anti-CD40L, or anti-CD80) or genetically engineering cells to reduce major histocompatibility complex (MHC) class I or increase immunosuppressive cytokine production, may also be explored to increase survival.[54]

To summarize, we describe a modified HDP system to generate large numbers of fully functional MΦs and use these cells to perform quantitative biodistribution studies. We found that liposomal clodronate pretreatment increases the in vivo survival of transplanted HDP-on cells from 1 to 7 days. Experiments in NCG mice demonstrated the survival and expansion of HDP-on cells to at least 14 days, demonstrating the role of B, T, and/or NK cells in preventing longer term survival. Our proposed model for this behavior is that HDP-on cells are removed by two overlapping mechanisms (Figure 7). The first is mediated by endogenous MΦs, which act within 7 days to remove transplanted cells. The second is mediated by B, T, and/or NK cells and removes transplanted cells beyond 7 days. Therefore, while liposomal clodronate and genetic modifications may enhance the acute survival of HDPs, further efforts must be made to reduce the impact of the humoral and cell-mediated immune responses to increase the long-term survival of transplanted HDPs.

MATERIALS AND METHODS

Cell Culture

Reagents were acquired from the University of California, San Francisco (UCSF) Cell Culture Facility (UCSF CCF), unless otherwise indicated. HDPs were cultured in RPMI-1640 (50 mM HEPES, 1% penicillin-streptomycin (PenStrep)/amphotericin B antibiotic/antimycotic, 1% GlutaMAX [GIBCO], 10% heat-inactivated fetal calf serum [Hyclone], 0.55 mM 2-mercaptoethanol [Life Technologies], 1% GMCSF supplement [see Supplemental Materials and Methods]), using tissue-culture-treated flasks from Grenier Bio-One (product #658170) in a humidified incubator maintained at 37°C and 5% CO_2. Cells expressing the constitutively active GMCSFR were cultured without the GMCSF supplement. Cell counts were determined using a hemocytometer, and live cells were enumerated using trypan blue staining.

HDPs and HDP-on cells were differentiated into MΦs using different protocols. MΦs derived from HDPs were differentiated by culturing

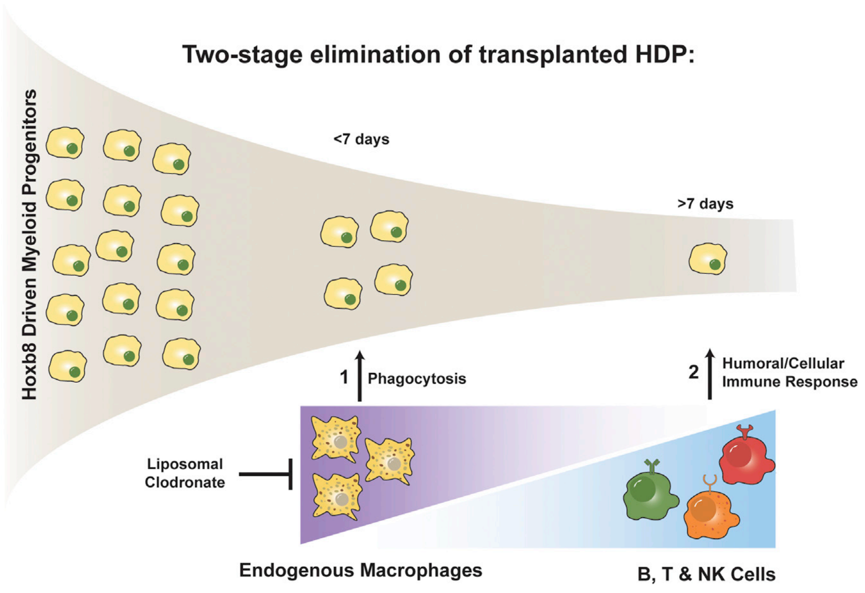

Figure 7. Model of Two-Stage Immune Rejection of HDPs

Removal of endogenous MΦs by liposomal clodronate increases acute survival, but humoral and cellular immune responses from B, T, and/or NK cells prevents long-lasting engraftment.

cells in 200 nM 4-OHT (Enzo Life Sciences) for 10 days. Differentiation was started with a cell density of 2–4 × 10^5 cells per milliliter, and the media were changed every 2 days for the first 6 days. Thereafter, no media changes were performed until MΦ were collected on day 10. MΦ differentiated from HDP-on cells were differentiated using 40 nM 4-OHT and 1 μM ruxolitinib (Selleckchem) for 6 days. Differentiation was started with a cell density of 2–4 × 10^5 cells per milliliter, and the media were changed only on day 3.

Plasmid Construction

Plasmids for lentivirus and retrovirus production were cloned using standard techniques, including restriction cloning and Gibson assembly, depending on the applicability of each technique to the desired product. For the production of plasmids containing Hoxb8 (NCBI Gene ID: 15416), GMCSFR (NCBI Gene ID: 12983), and IRF8 (NCBI Gene ID: 15900), murine cDNAs were acquired from GE Dharmacon and cloned into pLVX or pMSCV vectors for lentivirus or retrovirus production, respectively, as described in the following section. Expression constructs also encoded antibiotic selection markers: blasticidin (bsd), puromycin (puro), zeocin (zeo), and neomycin (neo). To engineer the constitutive activity of GMCSFR, a QuikChange Lightning kit (Agilent Technologies) was used to modify the leucine in position 452 to glutamic acid (L452E), as adapted from Perugini et al.[29] Vectors for plasmid construction were obtained from Addgene or commercially available from Clontech Laboratories. Constructs were sequence verified before use.

lin$^-$ Bone Marrow Culture and Generating Hoxb8-Dependent Progenitors

The lin$^-$ cells were collected from the bone marrow of healthy female BALB/c mice by purifying the cells using a lineage depletion kit (Miltenyi Biotec, #130-090-858) as per manufacturer protocols (see Supplemental Materials and Methods). Following collection, the lin$^-$ cells were cultured overnight in 100 ng/mL stem cell factor (SCF) (#250-03), 10 ng/mL IL-3 (#213-13), and 20 ng/mL IL-6 (#216-16) (all cytokines were murine and obtained from PeproTech) before they were transduced by lentivirus encoding the Hoxb8 construct. Following transduction with the Hoxb8 lentivirus, the cells were cultured in 30 ng/mL GMCSF (Peprotech, 315-03).

Lentivirus and Retrovirus Production

A second-generation lentivirus was used, requiring three plasmids: pCMV-dR8.91 (Delta 8.9) (containing gag, pol, and rev genes; Addgene #12263), VSV-G (envelope; Addgene #8454), and expression construct (pLVX-insert; Clontech #632187). For murine stem cell retrovirus, two plasmids are required: the packaging vector (pCL-Eco; Addgene #12371) and the expression construct (pMSCV; Clontech #634401). To produce either retroviral or lentiviral vectors, HEK293T cells were transfected using Lipofectamine 2000 (Life Technologies) with the required plasmids, and the media were collected and replaced every day for 3 days (see Supplemental Materials and Methods). The media containing the virus vector were concentrated by mixing 1:3 (v/v) with Lenti-X or Retro-X concentrator (Clontech) overnight and centrifugation at 3,000 rpm for 30 min in a 50-mL conical tube. The concentrated virus vector was resuspended in 200 μL RPMI and was used without further purification for viral vector transduction.

Lentiviral and Retroviral Transduction

Cells were transduced with retrovirus or lentivirus vectors using the Spinfection method, as described in the Supplemental Materials and Methods.[31] Briefly, 2 × 10^4 cells were added to a RetroNectin (Clontech #T100B)-treated 48-well plate along with 50 μL retrovirus or lentivirus vector. The plate was centrifuged for 90 min at 1,500 × *g* and 30°C. The culture was then allowed to recover overnight in a humidified 5% CO_2 32°C incubator. The following day, the culture was returned to a humidified 5% CO_2 37°C incubator. After 3–5 days, the culture was expanded and tested for integration of the desired modifications. For antibiotic selection, 6 μg/mL blasticidin, 0.2 μg/mL puromycin, 30 μg/mL zeocin, or 1 mg/mL neomycin (Life Technologies) was added to the media.

MΦ M1/M2 Polarization

MΦs were prepared as described previously. To polarize MΦs, the media were changed for the appropriate polarization media: 10 ng/mL LPS (Sigma), or 100 ng/mL IL-4 (Peprotech) for M1 or

M2, respectively. Following an overnight treatment, MΦs were washed with D-PBS, and RNA was collected using the RNeasy Mini Kit (QIAGEN) following manufacturer protocols. The expression of M1/M2 genes was then measured by qRT-PCR.

Real-Time qPCR

RNA was collected from cell samples as previously described. cDNA for real-time qPCR was produced using the SuperScript VILO cDNA Synthesis Kit (ThermoFisher). Real-time qPCR was performed using SsoFast EvaGreen Supermix (BioRad) following manufacturer protocols, using a BioRad CFX96 thermal cycler. Each readout was normalized against an internal mouse beta-actin expression value and then compared to matched genes in other samples to determine fold change. For each sample and each gene, three replicates were performed, and the fold enhancement was averaged to yield a single value. To generate multiple values for statistical analysis, multiple experimental samples, as indicated in the relevant figure, were subjected to the described method. For primer sequences, please refer to the Supplemental Materials and Methods.

Flow Cytometry

Flow cytometry was conducted at the UCSF Flow Cytometry core on a BD Fortessa instrument (see Supplemental Materials and Methods). The isotype antibody control used was APC-labeled Rat IgG2a, κ (BioLegend, Clone RTK2758). Fc-receptor blocking was performed using rat anti-mouse CD16/CD32 (BD PharMingen, Clone 2.4G2). Cells were labeled with allophycocyanin (APC)-labeled rat anti-F4/80 (BioLegend, Clone BM8, Rat IgG2a, κ) according to the manufacturer's instructions, and the data were analyzed using FlowJo software. Before analysis, dead cells and doublets were removed using forward scatter (FSC) and side scatter (SSC) gating.

MΦ Phagocytosis

Fluorescent liposomes were prepared with a 3:1:2 mole ratio of HSPC:DSPG:cholesterol (HSPC: L-α-phosphatidylcholine, hydrogenated (Soy); DSPG: 1,2-distearoyl-*sn*-glycero-3-phospho-(1'-*rac*-glycerol) [Avanti Polar Lipids]) with 1% 8-hydroxypyrene-1,3,6-trisulfonic acid (HPTS) (Sigma). The mixture of lipids in chloroform were placed in a round-bottom flask, and the chloroform was removed using a rotary evaporator. The resulting lipid film was dried under high vacuum overnight at room temperature. The film was reconstituted with HBS (140 mM NaCl, 10 mM HEPES) and sonicated under argon at room temperature for 40 min to form liposomes. The liposomes were then dialyzed for 24 hr in 2 L HBS in a 10,000-molecular-weight (MW) dialysis cassette (ThermoScientific) to remove unencapsulated HPTS and sterile filtered through a 0.45 μm filter (Millipore). For quantitative studies, liposomes were prepared in a similar fashion, using a 1:3:2 mole ratio of DSPG/DSPC/cholesterol (DSPC: 1,2-distearoyl-*sn*-glycero-3-phosphocholine) with 0.01% DiD (Biotium). Lipid films were prepared as described and sonicated with D-PBS under argon at 45°C for 20 min. DiD-labeled liposomes were extruded through a 100-nm polycarbonate membrane before sterile filtering through a 0.45 μm filter. The size and charge of the liposomes were determined using a Zetasizer (HPTS: diameter, 76 nm; PDI, 0.76; charge, −57 mV; DiD: diameter, 116 nm; PDI, 0.216; charge, −27.3 mV). Fluorescent bacteria were generated by transforming a BL21 *E. coli* strain with a pGEX-TagBFP plasmid.

Uptake experiments were performed on MΦs differentiated from HDPs treated with 200 nM 4-OHT for 10 days or HDP-on cells treated with 40 nM 4-OHT and 1 μM ruxolitinib for 6 days. MΦs were removed from T-75 tissue culture plates using 5 mL HyQTase (GE Healthcare) for 10 min, and 1×10^5 MΦs were plated overnight in 1 mL media in a 12-well plate prior to incubation with liposomes or bacteria. For fluorescent imaging, MΦs were incubated with 1 mL 500 μM HPTS-liposome solution or 1 mL 10% live TagBFP-*E. coli* culture (optical density 600 [OD_{600}] = 0.5) in serum-free media for 3 hr or 30 min, respectively, in a humidified 5% CO_2 37°C incubator. Following the incubations, wells were rinsed three times with 1 mL D-PBS. Cultures treated with bacteria were imaged without any further treatment, while liposome-treated cultures were stained with DAPI prior to imaging on a fluorescent microscope. For quantitative liposomal uptake studies, 1×10^5 MΦs were plated overnight in 1 mL media in a 12-well plate prior to incubation with liposomes. The next day, MΦs were incubated with DiD liposomes at varying concentrations in serum-free media for 6 hr in a humidified 5% CO_2 37°C incubator. The MΦ cell line, RAW264, was used as a comparative phagocytosis positive control cell line. The wells were washed with D-PBS three times, and then the cells were lysed with 1 mL radio immunoprecipitation assay (RIPA) buffer (150 mM NaCl [Sigma], 1% Triton-100 [Sigma], 10% glycerol [molecular grade; Roche], and 50 mM Tris [Fisher Scientific]). Total fluorescence was measured using a spectrofluorometer (Fluorlog; Horiba) (excitation, 644; emission, 665). The amount of liposomes taken up by the cells was determined from a standard curve of DiD liposomes in RIPA buffer. To normalize the fluorescence signal to the number of MΦs in the well, total protein was measured using a BCA Protein Assay Kit (ThermoFisher).

Animals

All mice used in this study were purchased from Charles River Laboratories (Wilmington, MA) and maintained under pathogen-free conditions at the University of California, San Francisco (UCSF). All mouse procedures were approved by the UCSF Institutional Animal Care and Use Committee (IACUC). Two mouse strains were used in this study: BALB/c (strain #028) and NCG (NOD-Prkdc^{em26Cd52}Il2rg^{em26Cd22}/NjuCrl; strain #572). Due to their immunodeficient status, NCG mice were housed in ultraclean barrier facilities.

Cell Transplantation

Adherent MΦs were removed from T-75 tissue culture flasks by treatment with 5 mL HyQTase (GE Healthcare) for 10 min and gentle tapping of the flask. After removal from the flask, the cells were treated identically to suspension cells. Suspension HDP cells were washed twice with plain RPMI buffer and counted, and the required dose was resuspended in 500 μL for intraperitoneal injection. For each biodistribution experiment, the luciferase activity per cell was

determined to calculate the total luciferase units injected into the mouse.

Mice that were treated with liposomal clodronate (ClodLip BV) were dosed twice (4 days and 1 day prior to cell injection) with 100 μL liposomal clodronate (5 mg/mL) i.p. After the injection, the mouse was placed in a cage and observed for 10 min to ensure no adverse effects. No adverse effects were ever observed during this observation period. However, as reported by other groups, there was a 20%–25% mortality within 5 days of liposomal clodronate treatment.[32] All animals were female and 8–10 weeks old at the time of injection.

Biodistribution

Animals were euthanized by an intraperitoneal injection of sodium pentobarbital (200 mg/kg) and cervical dislocation, as approved by UCSF IACUC. Organs were collected, weighed, and placed on ice. Each organ was lysed with RIPA buffer (~200 mg tissue per milliliter of RIPA buffer) using a glass dounce grinder with a tight-fitting pestle, and a lysate was formed by using the pestle until no tissue was visible. The organ lysates were centrifuged at 3,000 rpm for 5 min, and the supernatant was used for further analysis. Blood samples (~50–200 μL) were collected into a tube containing 10 μL of 1 mg/mL heparin in D-PBS (Alfa Aeser) and were not processed further. Flushes of the peritoneal cavity were collected by injecting 5 mL of plain RPMI medium into the peritoneal cavity of a euthanized mouse. The mouse abdomen was massaged slightly to ensure proper mixing in the peritoneal cavity before a cut was made into the abdomen to drain the fluid with suspended cells into a collection dish. This suspension was transferred to a 15-mL tube and stored on ice until ready for measurement. Immediately prior to the luciferase activity measurement of samples from the peritoneal cavity, the cell suspension was mixed thoroughly by inverting the tube several times.

Luciferase activity was determined using SteadyGLO (Promega), as per manufacturer protocols. Clarified organ lysate (100 μL) was mixed with 100 μL SteadyGLO in a glass tube. Total luminescence was measured using a luminometer (MGM Instruments) over a 10-s period. The luciferase activity from the measured sample was multiplied with an appropriate correction factor to determine the total luciferase activity in each organ. The percentage of injected cells in each organ was calculated by dividing the total luciferase activity in each organ by the total luciferase activity of the injected cells. In control experiments, the presence of organ lysate did not reduce the luciferase activity of a known number of cells by more than 10%, compared to cells in RIPA alone (data not shown).

Statistics

Statistics analysis was performed using GraphPad Prism 5. To determine the significance between datasets, one-way ANOVA was performed, followed by a Bonferroni post-test. For comparison of pharmacokinetic data, a one-sided t test was used to compare within the same time point. Significance was reported as follows: *$p < 0.05$, **$p < 0.01$, or ***$p < 0.001$. Error bars represent SE.

SUPPLEMENTAL INFORMATION

Supplemental Information includes Supplemental Materials and Methods and nine figures and can be found with this article online at http://dx.doi.org/10.1016/j.omtm.2017.08.007.

AUTHOR CONTRIBUTIONS

S.K. designed and performed experiments, designed the original cell-construct strategy, and analyzed data. S.L. designed and performed experiments, analyzed data, and wrote the paper. F.C.S. conceived the project and wrote the paper.

CONFLICTS OF INTEREST

The authors declare no conflicts of interest.

ACKNOWLEDGMENTS

We thank the Alliston lab at UCSF for equipment support, the UCSF flow cytometry core for assistance with flow cytometry experiments, and the Nikon Imaging Center at UCSF for imaging support. We would like to thank Heather Hamilos of Charles River Laboratories for the generous gift of the NCG mice. This work was supported by an NSERC PGS-D fellowship (to S.L.) and by the NIH/NCI (R21CA182703 to F.C.S.).

REFERENCES

1. Wang, G.G., Calvo, K.R., Pasillas, M.P., Sykes, D.B., Häcker, H., and Kamps, M.P. (2006). Quantitative production of macrophages or neutrophils ex vivo using conditional Hoxb8. Nat. Methods *3*, 287–293.
2. Condeelis, J., and Pollard, J.W. (2006). Macrophages: obligate partners for tumor cell migration, invasion, and metastasis. Cell *124*, 263–266.
3. Mass, E., Ballesteros, I., Farlik, M., Halbritter, F., Günther, P., Crozet, L., Jacome-Galarza, C.E., Händler, K., Klughammer, J., Kobayashi, Y., et al. (2016). Specification of tissue-resident macrophages during organogenesis. Science *353*, aaf4238.
4. Okabe, Y., and Medzhitov, R. (2016). Tissue biology perspective on macrophages. Nat. Immunol. *17*, 9–17.
5. Zhou, P., Shaffer, D.R., Alvarez Arias, D.A., Nakazaki, Y., Pos, W., Torres, A.J., Cremasco, V., Dougan, S.K., Cowley, G.S., Elpek, K., et al. (2014). In vivo discovery of immunotherapy targets in the tumour microenvironment. Nature *506*, 52–57.
6. Murray, P.J., Allen, J.E., Biswas, S.K., Fisher, E.A., Gilroy, D.W., Goerdt, S., Gordon, S., Hamilton, J.A., Ivashkiv, L.B., Lawrence, T., et al. (2014). Macrophage activation and polarization: nomenclature and experimental guidelines. Immunity *41*, 14–20.
7. Martinez, F.O., Gordon, S., Locati, M., and Mantovani, A. (2006). Transcriptional profiling of the human monocyte-to-macrophage differentiation and polarization: new molecules and patterns of gene expression. J. Immunol. *177*, 7303–7311.
8. Wynn, T.A., Chawla, A., and Pollard, J.W. (2013). Macrophage biology in development, homeostasis and disease. Nature *496*, 445–455.
9. Haldar, M., Kohyama, M., So, A.Y., Kc, W., Wu, X., Briseño, C.G., Satpathy, A.T., Kretzer, N.M., Arase, H., Rajasekaran, N.S., et al. (2014). Heme-mediated SPI-C induction promotes monocyte differentiation into iron-recycling macrophages. Cell *156*, 1223–1234.
10. Sato, K. (2015). Effects of microglia on neurogenesis. Glia *63*, 1394–1405.
11. Zhao, Y., Haney, M.J., Gupta, R., Bohnsack, J.P., He, Z., Kabanov, A.V., and Batrakova, E.V. (2014). GDNF-transfected macrophages produce potent neuroprotective effects in Parkinson's disease mouse model. PLoS ONE *9*, e106867.

12. Escobar, G., Moi, D., Ranghetti, A., Ozkal-Baydin, P., Squadrito, M.L., Kajaste-Rudnitski, A., Bondanza, A., Gentner, B., De Palma, M., Mazzieri, R., and Naldini, L. (2014). Genetic engineering of hematopoiesis for targeted IFN-α delivery inhibits breast cancer progression. Sci. Transl. Med. *6*, 217ra3.

13. Kan, O., Day, D., Iqball, S., Burke, F., Grimshaw, M.J., Naylor, S., and Binley, K. (2011). Genetically modified macrophages expressing hypoxia regulated cytochrome P450 and P450 reductase for the treatment of cancer. Int. J. Mol. Med. *27*, 173–180.

14. Burke, B. (2003). Macrophages as novel cellular vehicles for gene therapy. Expert Opin. Biol. Ther. *3*, 919–924.

15. Zhao, Y., Haney, M.J., Mahajan, V., Reiner, B.C., Dunaevsky, A., Mosley, R.L., Kabanov, A.V., Gendelman, H.E., and Batrakova, E.V. (2011). Active targeted macrophage-mediated delivery of catalase to affected brain regions in models of Parkinson's disease. J. Nanomed. Nanotechnol. *S4*, 003.

16. Choi, J., Kim, H.Y., Ju, E.J., Jung, J., Park, J., Chung, H.K., Lee, J.S., Lee, J.S., Park, H.J., Song, S.Y., et al. (2012). Use of macrophages to deliver therapeutic and imaging contrast agents to tumors. Biomaterials *33*, 4195–4203.

17. Batrakova, E.V., Gendelman, H.E., and Kabanov, A.V. (2011). Cell-mediated drug delivery. Expert Opin. Drug Deliv. *8*, 415–433.

18. Dou, H., Grotepas, C.B., McMillan, J.M., Destache, C.J., Chaubal, M., Werling, J., Kipp, J., Rabinow, B., and Gendelman, H.E. (2009). Macrophage delivery of nanoformulated antiretroviral drug to the brain in a murine model of neuroAIDS. J. Immunol. *183*, 661–669.

19. Lee, S., Kivimae, S., Dolor, A., and Szoka, F.C. (2016). Macrophage-based cell therapies: the long and winding road. J. Control. Release *240*, 527–540.

20. Norelli, M., Casucci, M., Bonini, C., and Bondanza, A. (2016). Clinical pharmacology of CAR-T cells: Linking cellular pharmacodynamics to pharmacokinetics and antitumor effects. Biochim. Biophys. Acta *1865*, 90–100.

21. Trounson, A., and McDonald, C. (2015). Stem cell therapies in clinical trials: progress and challenges. Cell Stem Cell *17*, 11–22.

22. Staal, F.J., Baum, C., Cowan, C., Dzierzak, E., Hacein-Bey-Abina, S., Karlsson, S., Lapidot, T., Lemischka, I., Mendez-Ferrer, S., Mikkers, H., et al. (2011). Stem cell self-renewal: lessons from bone marrow, gut and iPS toward clinical applications. Leukemia *25*, 1095–1102.

23. Stevenson, H.C., Foon, K.A., and Sugarbaker, P.H. (1986). Ex vivo activated monocytes and adoptive immunotherapy trials in colon cancer patients. Prog. Clin. Biol. Res. *211*, 75–82.

24. Andreesen, R., Scheibenbogen, C., Brugger, W., Krause, S., Meerpohl, H.G., Leser, H.G., Engler, H., and Löhr, G.W. (1990). Adoptive transfer of tumor cytotoxic macrophages generated in vitro from circulating blood monocytes: a new approach to cancer immunotherapy. Cancer Res. *50*, 7450–7456.

25. Haney, M.J., Zhao, Y., Harrison, E.B., Mahajan, V., Ahmed, S., He, Z., Suresh, P., Hingtgen, S.D., Klyachko, N.L., Mosley, R.L., et al. (2013). Specific transfection of inflamed brain by macrophages: a new therapeutic strategy for neurodegenerative diseases. PLoS ONE *8*, e61852.

26. Aziz, A., Soucie, E., Sarrazin, S., and Sieweke, M.H. (2009). MafB/c-Maf deficiency enables self-renewal of differentiated functional macrophages. Science *326*, 867–871.

27. Redecke, V., Wu, R., Zhou, J., Finkelstein, D., Chaturvedi, V., High, A.A., and Häcker, H. (2013). Hematopoietic progenitor cell lines with myeloid and lymphoid potential. Nat. Methods *10*, 795–803.

28. Rosas, M., Osorio, F., Robinson, M.J., Davies, L.C., Dierkes, N., Jones, S.A., Reis e Sousa, C., and Taylor, P.R. (2011). Hoxb8 conditionally immortalised macrophage lines model inflammatory monocytic cells with important similarity to dendritic cells. Eur. J. Immunol. *41*, 356–365.

29. Perugini, M., Brown, A.L., Salerno, D.G., Booker, G.W., Stojkoski, C., Hercus, T.R., Lopez, A.F., Hibbs, M.L., Gonda, T.J., and D'Andrea, R.J. (2010). Alternative modes of GM-CSF receptor activation revealed using activated mutants of the common beta-subunit. Blood *115*, 3346–3353.

30. Rosenbauer, F., and Tenen, D.G. (2007). Transcription factors in myeloid development: balancing differentiation with transformation. Nat. Rev. Immunol. *7*, 105–117.

31. Berggren, W.T., Lutz, M., and Modesto, V. (2008). General Spinfection Protocol (StemBook).

32. Li, Z., Xu, X., Feng, X., and Murphy, P.M. (2016). The macrophage-depleting agent clodronate promotes durable hematopoietic chimerism and donor-specific skin allograft tolerance in mice. Sci. Rep. *6*, 22143.

33. Wurdinger, T., Badr, C., Pike, L., de Kleine, R., Weissleder, R., Breakefield, X.O., and Tannous, B.A. (2008). A secreted luciferase for ex vivo monitoring of in vivo processes. Nat. Methods *5*, 171–173.

34. Suzuki, T., Arumugam, P., Sakagami, T., Lachmann, N., Chalk, C., Sallese, A., Abe, S., Trapnell, C., Carey, B., Moritz, T., et al. (2014). Pulmonary macrophage transplantation therapy. Nature *514*, 450–454.

35. Mosser, D.M., and Edwards, J.P. (2008). Exploring the full spectrum of macrophage activation. Nat. Rev. Immunol. *8*, 958–969.

36. Bressani, R.F., Nowacek, A.S., Singh, S., Balkundi, S., Rabinow, B., McMillan, J., Gendelman, H.E., and Kanmogne, G.D. (2011). Pharmacotoxicology of monocyte-macrophage nanoformulated antiretroviral drug uptake and carriage. Nanotoxicology *5*, 592–605.

37. Nowacek, A.S., Miller, R.L., McMillan, J., Kanmogne, G., Kanmogne, M., Mosley, R.L., Ma, Z., Graham, S., Chaubal, M., Werling, J., et al. (2009). NanoART synthesis, characterization, uptake, release and toxicology for human monocyte-macrophage drug delivery. Nanomedicine (Lond.) *4*, 903–917.

38. Thompson, H.L., van Rooijen, N., McLelland, B.T., and Manilay, J.O. (2016). F4/80(+) host macrophages are a barrier to murine embryonic stem cell-derived hematopoietic progenitor engraftment in vivo. J. Immunol. Res. *2016*, 2414906.

39. Happle, C., Lachmann, N., Škuljec, J., Wetzke, M., Ackermann, M., Brennig, S., Mucci, A., Jirmo, A.C., Groos, S., Mirenska, A., et al. (2014). Pulmonary transplantation of macrophage progenitors as effective and long-lasting therapy for hereditary pulmonary alveolar proteinosis. Sci. Transl. Med. *6*, 250ra113.

40. van Rooijen, N., and Hendrikx, E. (2010). Liposomes for specific depletion of macrophages from organs and tissues. Methods Mol. Biol. *605*, 189–203.

42. Robbins, C.S., Hilgendorf, I., Weber, G.F., Theurl, I., Iwamoto, Y., Figueiredo, J.L., Gorbatov, R., Sukhova, G.K., Gerhardt, L.M., Smyth, D., et al. (2013). Local proliferation dominates lesional macrophage accumulation in atherosclerosis. Nat. Med. *19*, 1166–1172.

43. Boulter, L., Govaere, O., Bird, T.G., Radulescu, S., Ramachandran, P., Pellicoro, A., Ridgway, R.A., Seo, S.S., Spee, B., Van Rooijen, N., et al. (2012). Macrophage-derived Wnt opposes Notch signaling to specify hepatic progenitor cell fate in chronic liver disease. Nat. Med. *18*, 572–579.

44. van Rooijen, N., Kors, N., and Kraal, G. (1989). Macrophage subset repopulation in the spleen: differential kinetics after liposome-mediated elimination. J. Leukoc. Biol. *45*, 97–104.

45. Van Rooijen, N., Kors, N., vd Ende, M., and Dijkstra, C.D. (1990). Depletion and repopulation of macrophages in spleen and liver of rat after intravenous treatment with liposome-encapsulated dichloromethylene diphosphonate. Cell Tissue Res. *260*, 215–222.

46. Buiting, A.M., Zhou, F., Bakker, J.A., van Rooijen, N., and Huang, L. (1996). Biodistribution of clodronate and liposomes used in the liposome mediated macrophage 'suicide' approach. J. Immunol. Methods *192*, 55–62.

47. Ansari, A.M., Ahmed, A.K., Matsangos, A.E., Lay, F., Born, L.J., Marti, G., Harmon, J.W., and Sun, Z. (2016). Cellular GFP toxicity and immunogenicity: potential confounders in in vivo cell tracking experiments. Stem Cell Rev. *12*, 553–559.

48. Stripecke, R., Carmen Villacres, M., Skelton, D., Satake, N., Halene, S., and Kohn, D. (1999). Immune response to green fluorescent protein: implications for gene therapy. Gene Ther. *6*, 1305–1312.

49. Riddell, S.R., Elliott, M., Lewinsohn, D.A., Gilbert, M.J., Wilson, L., Manley, S.A., Lupton, S.D., Overell, R.W., Reynolds, T.C., Corey, L., and Greenberg, P.D. (1996). T-cell mediated rejection of gene-modified HIV-specific cytotoxic T lymphocytes in HIV-infected patients. Nat. Med. *2*, 216–223.

50. Tabayoyong, W.B., Salas, J.G., Bonde, S., and Zavazava, N. (2009). HOXB4-transduced embryonic stem cell-derived Lin-c-kit+ and Lin-Sca-1+ hematopoietic progenitors express H60 and are targeted by NK cells. J. Immunol. *183*, 5449–5457.

51. Swijnenburg, R.J., Schrepfer, S., Govaert, J.A., Cao, F., Ransohoff, K., Sheikh, A.Y., Haddad, M., Connolly, A.J., Davis, M.M., Robbins, R.C., and Wu, J.C. (2008). Immunosuppressive therapy mitigates immunological rejection of human embryonic stem cell xenografts. Proc. Natl. Acad. Sci. USA *105*, 12991–12996.

52. Swijnenburg, R.J., Schrepfer, S., Cao, F., Pearl, J.I., Xie, X., Connolly, A.J., Robbins, R.C., and Wu, J.C. (2008). In vivo imaging of embryonic stem cells reveals patterns of survival and immune rejection following transplantation. Stem Cells Dev. *17*, 1023–1029.

53. Huber, B.C., Ransohoff, J.D., Ransohoff, K.J., Riegler, J., Ebert, A., Kodo, K., Gong, Y., Sanchez-Freire, V., Dey, D., Kooreman, N.G., et al. (2013). Costimulation-adhesion blockade is superior to cyclosporine A and prednisone immunosuppressive therapy for preventing rejection of differentiated human embryonic stem cells following transplantation. Stem Cells *31*, 2354–2363.

54. Pearl, J.I., Kean, L.S., Davis, M.M., and Wu, J.C. (2012). Pluripotent stem cells: immune to the immune system? Sci. Transl. Med. *4*, 164ps25.

OMTM, Volume 7

Supplemental Information

Clodronate Improves Survival of Transplanted Hoxb8 Myeloid Progenitors with Constitutively Active GMCSFR in Immunocompetent Mice

Simon Lee, Saul Kivimäe, and Francis C. Szoka

1 Supplemental Figures

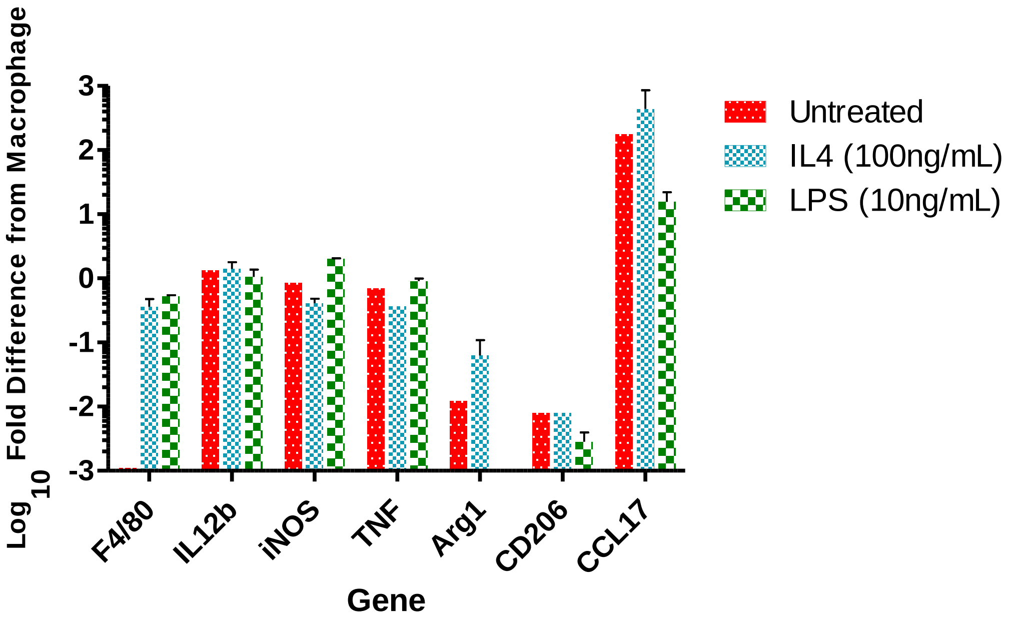

Figure S1: Gene expression of HDP-on treated with M1 and M2 inducers. HDP-on were treated overnight with 10 ng/mL LPS or 100 ng/mL IL-4 and gene expression of established M1 and M2 genes (M1: IL12b, iNOS, TNF; M2: Arg1, CD206, CCL17) was measured by qPCR. Fold enhancement is expressed relative to untreated HDP-on MΦs.

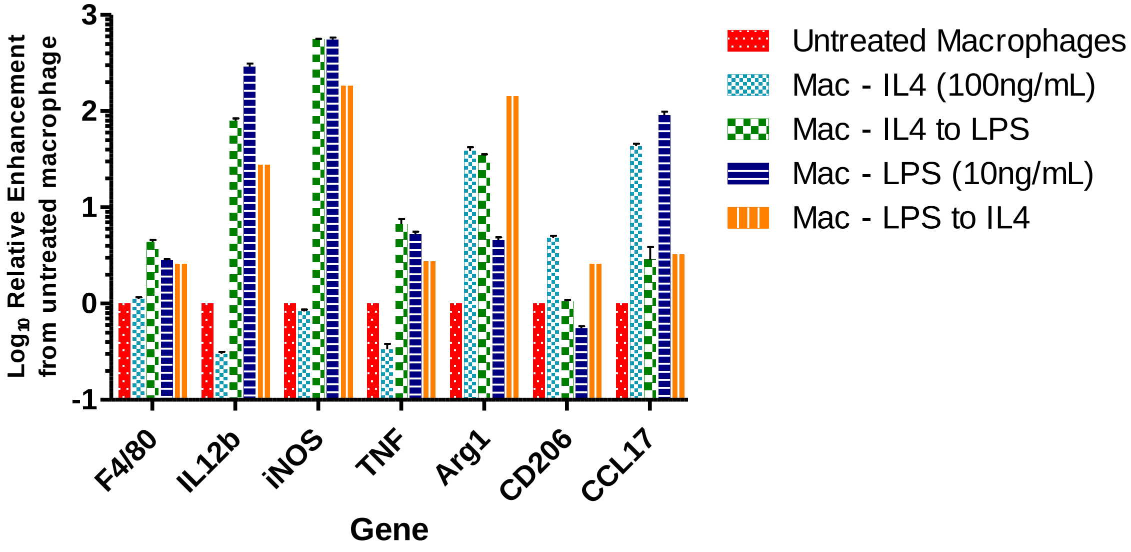

Figure S2: Relative gene expression of MΦ and M1/M2 markers of M1/M2 polarized HDP-on MΦ treated with opposing M1/M2 inducers. Cultures were treated for 24 h with 100 ng/mL IL-4 or 10 ng/mL LPS. For IL-4 to LPS or LPS to IL-4 cultures, cells were treated for 24 h with 100 ng/mL IL-4 or 10 ng/mL LPS before the media was swapped for the opposing treatment for another 24 h. MΦ were differentiated for 6 days in 40 nM 4-OHT and 1 µM ruxolitinib in tissue culture plastic flasks before $1x10^5$ cells were plated in a 12 well plate overnight before treatment. Relative expression is calculated by comparing expression levels between differentiated cells and HDP-on MΦ.

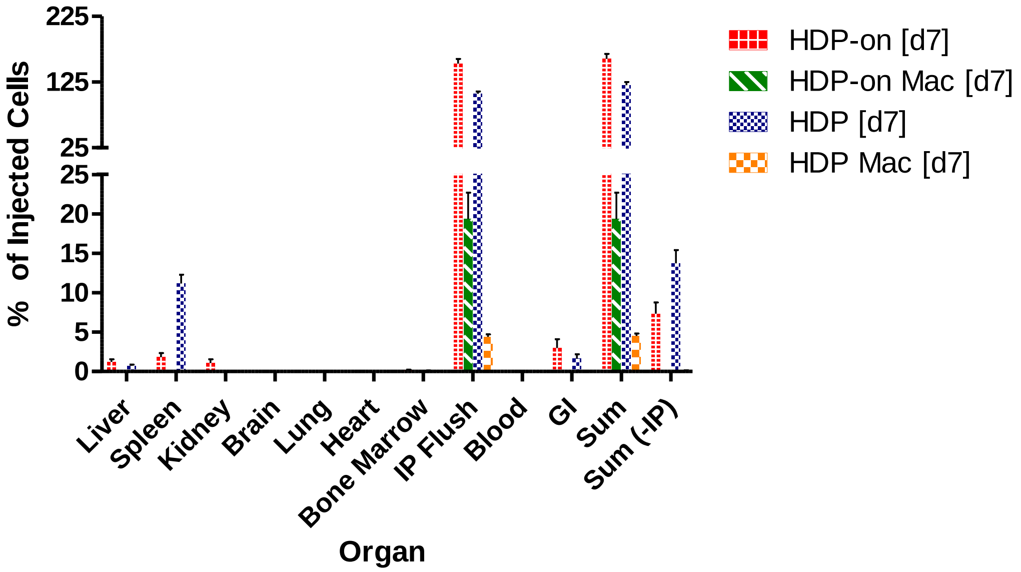

Figure S3: Biodistribution of HDP, HDP-on, HDP MΦs and HDP-on MΦs in immunodeficient NCG mice 7 days post injection. Mice were injected intraperitoneally with $5x10^6$ cells in 500 µL RPMI with a 28 gauge syringe, euthanized after 7 days and tissues were analyzed for luciferase activity. Statistics: N = 6 for HDP-on, N = 3 for other conditions.

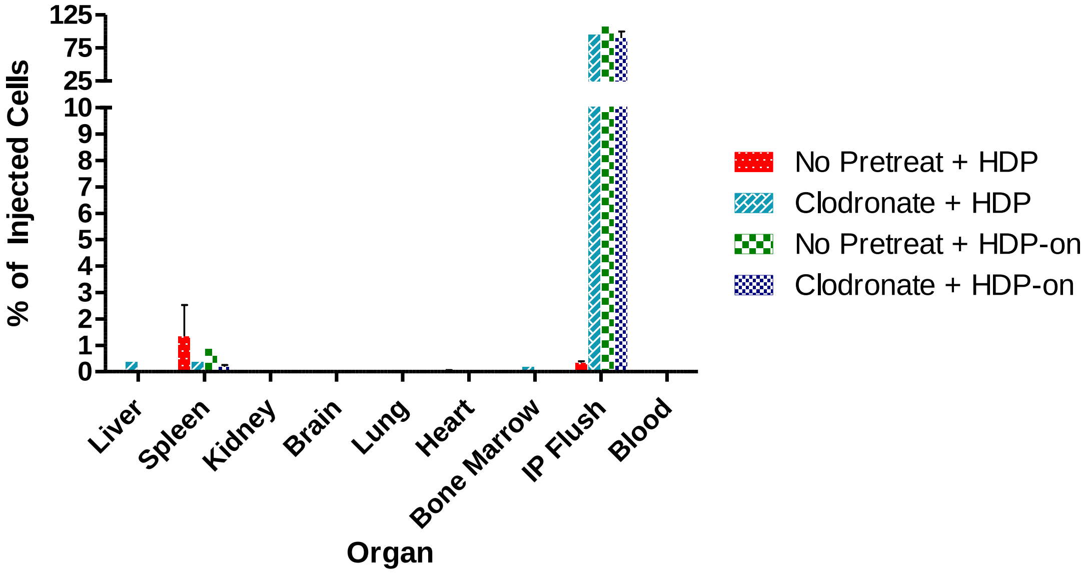

Figure S4: Biodistribution of HDP and HDP-on in mice pretreated with liposomal clodronate 1 day post-transplantation. For clodronate pretreatment, mice were injected IP with 100 µL liposomal clodronate (5mg/mL) 4 and 1 days before cell injection. Healthy BALB/c mice were injected intraperitoneally with $5x10^6$ cells in 500 µL RPMI with a 28 gauge syringe, euthanized after 1 days and tissues were analyzed for luciferase activity. Statistics: N = 3 per condition

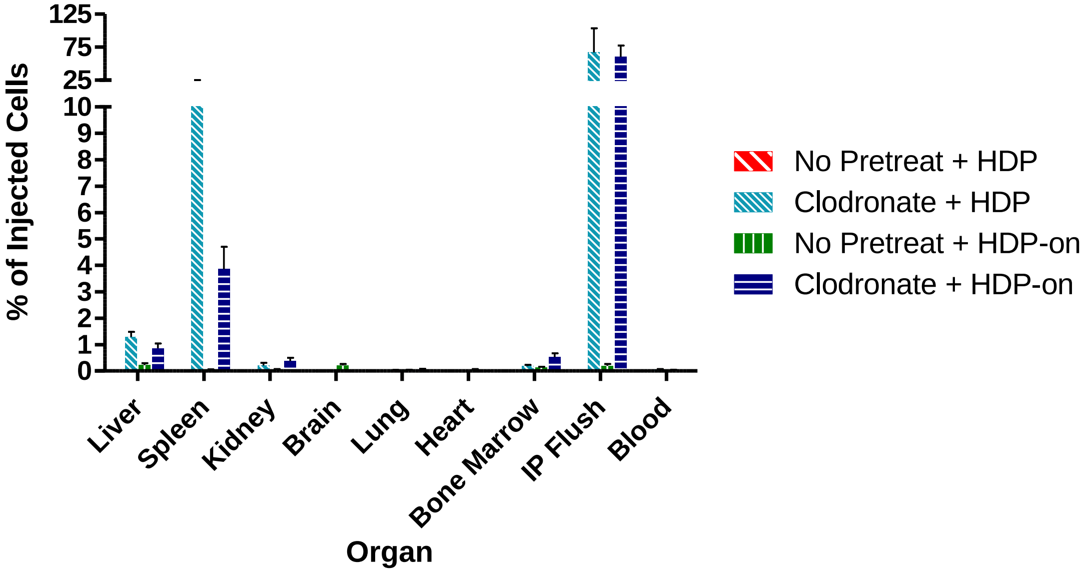

Figure S5: Biodistribution of HDP and HDP-on in mice pretreated with liposomal clodronate 7 days post-transplantation. For clodronate pretreatment, mice were injected IP with 100 µL liposomal clodronate (5mg/mL) 4 and 1 days before cell injection. Healthy BALB/c mice were injected intraperitoneally with $5x10^6$ cells in 500 µL RPMI with a 28

gauge syringe, euthanized after 7 days and tissues were analyzed for luciferase activity. Statistics: N = 3 for HDP with no liposomal clodronate, N = 6 for all other conditions

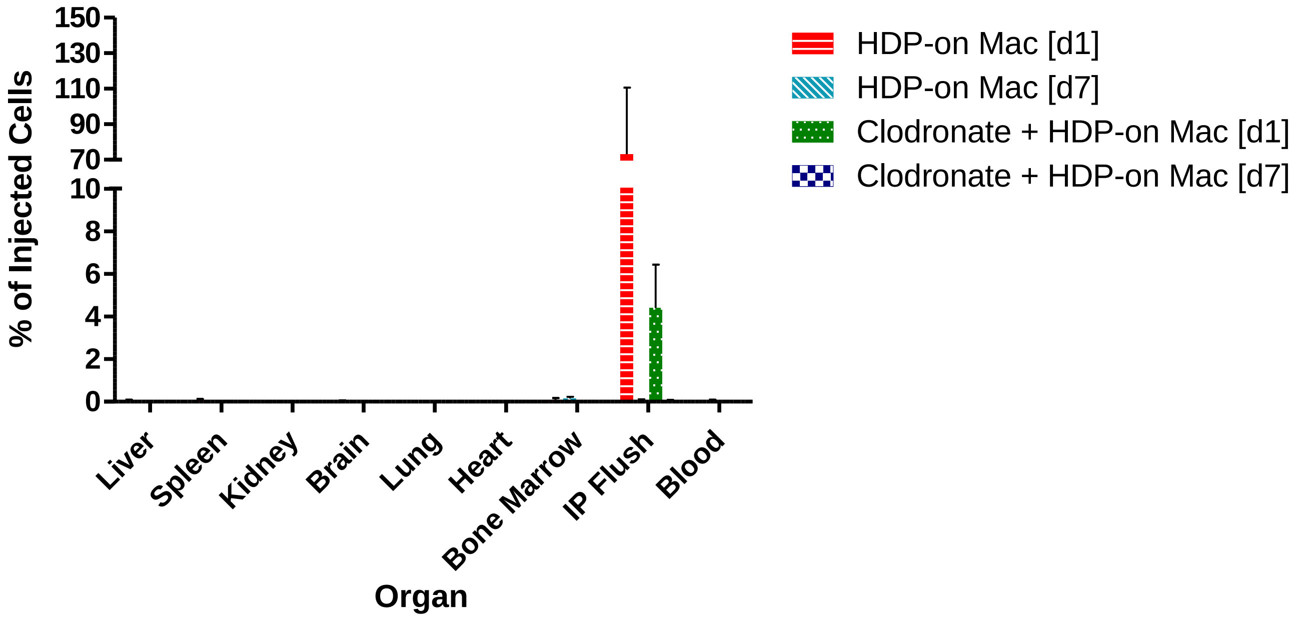

Figure S6: Biodistribution of macrophages derived from HDP and HDP-on in mice pretreated with liposomal clodronate 1 and 7 days post-transplantation. Healthy BALB/c mice were injected intraperitoneally with $5x10^6$ cells in 500 µL RPMI with a 28 gauge syringe, euthanized after 1 or 7 days and tissues were analyzed for luciferase activity. Statistics: N = 3 for all conditions

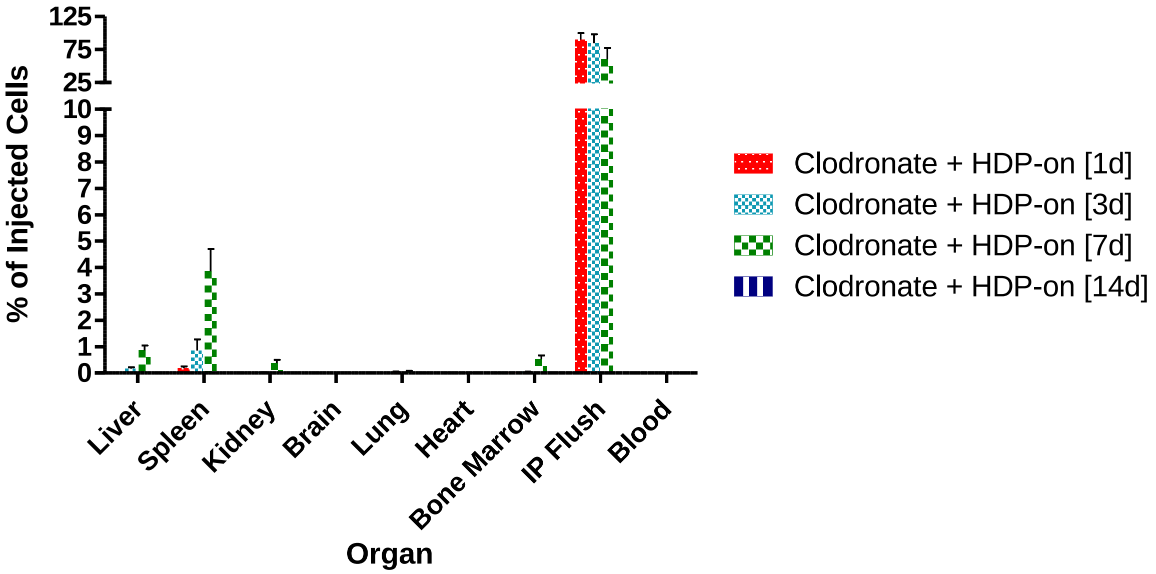

Figure S7: Biodistribution of HDP-on in clodronate-pretreated BALB/c mice at 1, 3, 7 and 14 days post-transplantation. Mice were injected IP with 100 µL liposomal clodronate (5mg/mL) 4 and 1 days before cell injection. BALB/c mice were injected intraperitoneally

with 5x10^6 cells in 500 µL RPMI with a 28 gauge syringe, euthanized after 14 days and tissues were analyzed for luciferase activity. Statistics: N = 3 per time point

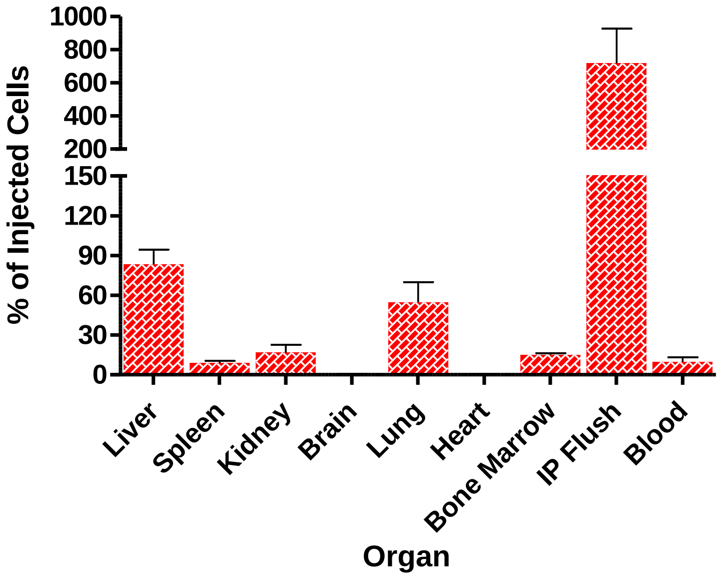

Figure S8: Biodistribution of HDP-on in immunodeficient NCG mice 14 days post-transplantation. NCG mice were injected intraperitoneally with 5x10^6 cells in 500 µL RPMI with a 28 gauge syringe, euthanized after 14 days and tissues were analyzed for luciferase activity. Statistics: N = 3

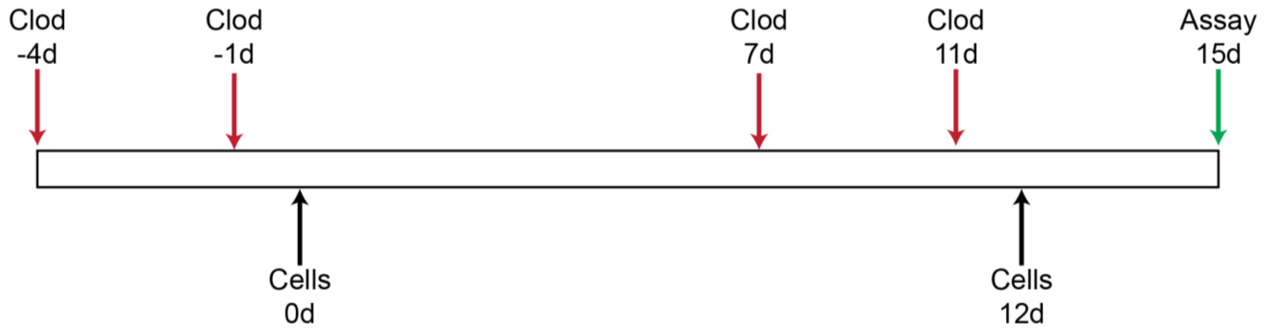

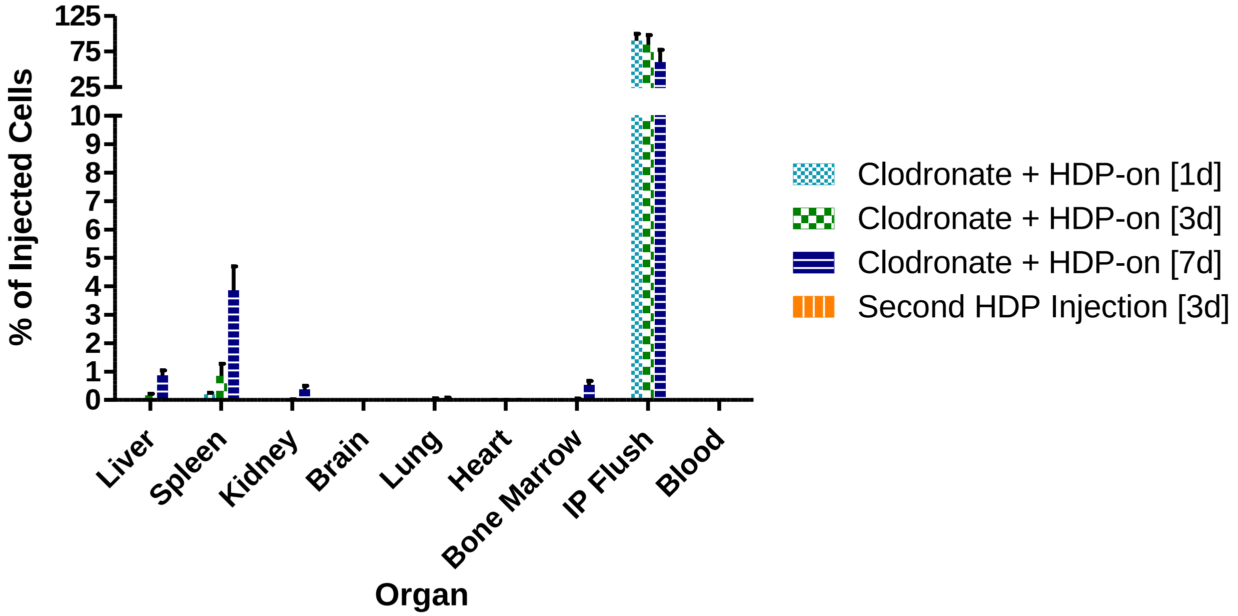

Figure S9: Biodistribution of HDP-on in clodronate-pretreated BALB/c mice which received a second injection of HDP-on. (Top): Treatment scheme of mice which received second injection. (Bottom): Biodistribution of mice which received second injection, presented with data from mice which receive only a single injection of HDP-on. Mice were injected IP with 100 µL liposomal clodronate (5 mg/mL) 4 and 1 days before cell injection. BALB/c mice were injected intraperitoneally with 5x10^{6} cells in 500 µL RPMI with a 28 gauge syringe, euthanized after 14 days and tissues were analyzed for luciferase activity. Statistics: N = 3 per time point

2 Methods

2.1 Supplemental GM-CSF Media

A GM-CSF expressing L929 cell line was generated by transducing L929 cells with a lentiviral construct (pLVX-GMCSF-IRES-tdTomato). Positively transduced cells were isolated by FACS and used to generate the supplemental media. GM-CSF L929 cells were grown to confluency, whereupon the media was switched to a low serum formulation (DMEM, 50 mM HEPES, 1% PenStrep/Amphotericin B antibiotic/antimycotic, 1% Glutamax (Gibco), 0.5% heat inactivated fetal calf serum (Hyclone)). This reduces the proliferation capacity of the cells but maintains survival. After 3 days, the media was collected, centrifuged, filtered and frozen for future use. GM-CSF levels were measured following the protocols from a GM-CSF ELISA kit from Boster.

2.2 Lin$^-$ bone marrow culture and infection

Preparation:

Progenitor outgrowth media:

RPMI + 10% FBS + 50 µM 2-mercaptoethanol + 100 ng/mL SCF + 10 ng/mL IL-3 + 20 ng/mL IL-6 (all cytokines from Peprotech)

Maintenance media:

RPMI1640 + 10% FBS +50 µM 2-mercaptoethanol + cytokine (30 ng/mL GM-CSF)

Method:

1. CO_2 euthanize one mouse and collect the leg bones: femur, tibia and fibula. Remove as much of the tissue as possible and rinse in PBS.
2. Crush bones in PBS/0.5%BSA+2% mouse serum 2x(1 wet crush + 1 dry crush), collect into 50 mL Falcon tube
3. Triturate cell clumps by pipetting up and down with 5 mL tissue culture pipet
4. Filter cells/bone fragments through 40 µm strainer into another 50 ml Falcon tube
5. Spin 5 min at 1500 rpm
6. Resuspend in 4 mL PBS/0.5%BSA
7. Load on 3 mL Ficoll-Paque gradient
8. Spin 10 min at 2000 rpm
9. Collect all cells except bottom pellet
10. Dilute in 43 mL PBS/0.5%BSA
11. Spin 5 min at 2000 rpm
12. Resuspend in 3 mL PBS/0.5%BSA
13. Count cells
14. Spin 5 min at 1500 rpm
15. Resuspend at 40 µl/10^7 cells in PBS/0.5%BSA
16. Add 10 µl of biotinylated antibody cocktail/ 10^7 cells (Miltenyi Lineage Depletion Kit)
17. Mix, incubate 20 min at 4°C in coldroom (mix gently second time at 10 min)
18. Add 30µl of PBS/0.5%BSA per 10^7 cells
19. Add 20µl of magnetic beads/ 10^7 cells (Miltenyi Lineage Depletion Kit)
20. Mix, incubate 15min at 4°C (mix gently second time at 7 min)
21. Add 1 mL PBS/0.5%BSA
22. Spin 10 min at 300g

23. Equilibrate Miltenyi MS column on magnet with 500 µl PBS/0.5%BSA while cells are spinning
24. Resuspend cells in 500 µl PBS/0.5%BSA per 10^8 cells
25. Apply cells to column
26. Collect flowthrough – **this contains the lin⁻ cells** (approximately $1x10^5$-$5x10^5$ cells depending on strain and age)
27. Wash column 2x with 750 µL PBS/0.5%BSA
28. Collect and pool washes with flowthru (total 2 mL)
29. Count cells
30. Spin 5 min at 1500rpm
31. Resuspend at 10^6 cells/ml in *progenitor outgrowth* media in 48 or 24 well (will grow/differentiate faster when more dense)
32. Incubate cells 24-48 h at 37°C
33. Count cells
34. Infect 2 x 10^5 cells/ml by spinoculation on retronectin coated 48 well plate in 0.3 mL at 3000rpm for 90min (*maintenance* media + conc. virus + 0.1% Lipofectamine2000)
35. Add 0.3 mL media after spin
36. Incubate overnight at 32°C in tissue culture incubator
37. Change ½ media next day
38. Passage non-adherent cells to new well with new media every 1-3 days

2.3 <u>Lentivirus and Retrovirus Production</u>

<u>Preparation</u>:

1. Culture HEK293T cells for at least 2 passages (~5 days) prior to transfection
3. Note: HEK293T cells have very poor adherency, especially during the virus production phase. Take great care in gently changing media, and use 1% gelatin treated flasks.

<u>Method</u>:

1. One day prior to transfection, passage HEK293T into a T75 treated with 1% gelatin solution (apply solution for ~10 mins, wash 2x with PBS) such that it will be 70-80% confluency on the day of transfection.
2. Prepare lipofectamine and DNA solution:
    a. 1.875 mL Opti-Mem + 75 µLLipofectamine 2000 (Invitrogen)
    b. 1.875 mL Opti-Mem + 30 µg DNA (Lentirivrus: 14 µg pLVX-insert, 6 µg VSV, 10 µg dR8.2; retrovirus: 15 µg pCL-Eco, 15 µg pMSCV-insert)
3. Allow solutions to incubate individually for 5 mins, then mix together, incubating the mixed solution for 20 mins.
4. Remove media from flask and add lipo/DNA solution (dilute to 10 mL Opti-MEM)
5. After 6 h, replace with fresh DMEM media.
6. Collect and change media every 24 h, up to 4 days, storing at 4°C.
    a. Check after 24 h for fluorescence of HEK293T cells if insertion construct has a fluorescent marker
    b. Be very careful, as the culture gets older, the cells become less adherent. Pipette gently and handle the flask with care to prevent the cells from sloughing off the surface

c. *All materials from this point on should be treated with bleach solution*

7. Filter the collected media through a 0.45 µm filter.
8. Add Lenti-X-Concentrator or Retro-X-Concentrator (Clontech) (~13 mL to 40 mL of media), and incubate overnight at 4C
9. Spin at max speed (1500 g) for 45 min at 4C, and resuspend pellet in 400 µL of PBS.
10. Aliquot into 50 or 100 µL vials and freeze at -80°C until ready for use.

2.4 <u>Lentivirus and Retrovirus Transduction</u>

1. Coat infection wells (48 well plate) day before with 5-10 ug/mL retronectin (Clontech) in PBS overnight at 4°C.
2. Next day rinse coated wells 1x with PBS
3. Block non-specific binding with PBS+0.5% BSA for 30 min at room temperature.
4. Rinse blocked wells 2x with cell media
5. Mix in coated wells 20,000 cells in 200 µL growth media + 50 µL virus + 0.1% Lipofectamine 2000
6. Spin plate for 90 min at 1500 g at 30°C.
7. After spin add 300 µL growth media
8. Place cells overnight to 32°C cell culture incubator
9. Transfer next day to 37°C
10. Expand and assay for integration 5 days after infection. Split before if too dense after 3-4 days.

2.5 <u>qPCR</u>

*Note that all pipette tips used in these protocols should be filter tipped to prevent cross contamination

Reverse transcription to generate first-strand cDNA

1. Mix the following components:
   a. 4 µL Superscript VILO Mastermix (Invitrogen)
   b. 500 ng RNA
   c. X µL DEPC-treated water to 20 µL final volume
2. In a thermocycler, using the following program:
   a. 25°C for 10 min
   b. 42°C for 60 min
   c. 85°C for 5 min
3. Dilute the 20 µL solution into 380 µL water and store at -20°C until ready for use

Quantitative PCR (qPCR)

1. For each single run, prepare the following mixture. A mastermix can be made without the cDNA and pipetted into the wells of the plate (96 well thin walled hard shell PCR plates HSP9655 (Bio-Rad)). cDNA should be added individually to each well (scale as appropriate):
   a. 10 µL SsoFast Evogreen MM (Bio-Rad)
   b. 1 µL cDNA
   c. 0.8 µL Forward Primer (10 µM)
   d. 0.8 µL Reverse Primer (10 µM)
   e. 7.4 µL Water

2. Prepare triplicates for each gene per each cDNA sample.
3. Seal the plate with B seals (MSB1001, Bio-Rad)
4. In a Bio-Rad CFX96 thermocycler, set the following program:

Cycling Step	Temperature (°C)	Time (s)	# of Cycles
Enzyme Activation	95	30	1
Denaturation	95	15	40
Annealing	59	20	
Extension	72	40	
Melt Curve	65-95 (0.5 inc)	5/step	1

5. For each sample, calculate the ΔC_t values between actin and the gene of interest. To determine fold change from an untreated sample, use the following equation:

$$Fold\ Change = 2^{-(\Delta C_{t-sample} - \Delta C_{t-control})}$$

qPCR Primers

Gene	Sequence	PrimerBank ID*
Actin F	GGCTGTATTCCCCTCCATCG	6671509a1
Actin R	CCAGTTGGTAACAATGCCATGT	
Elane F	AGCAGTCCATTGTGTGAACGG	7657060a1
Elane R	CACAGCCTCCTCGGATGAAG	
Prtn3 F	ATGGCTGGAAGCTACCCATC	31981542a1
Prtn3 R	TGCCCACCTACAATCTTGGAG	
Ms4a3 F	GTGGTTCTGTTTATCAGCCCTT	18875420a1
Ms4a3 R	ACAGTGGGTAGCCTGTGTAGA	
Plac8 F	GCTCAGGCACCAACAGTTATC	21105853a1
Plac8 R	GCTGCCACTTGACATCCAAGA	
Emr1 (F4/80) F	TGACTCACCTTGTGGTCCTAA	2078508a1
Emr1 (F4/80) R	CTTCCCAGAATCCAGTCTTTCC	
IL12b F	TGGTTTGCCATCGTTTTGCTG	6680397a1
IL12b R	ACAGGTGAGGTTCACTGTTTCT	
iNOS (Nos 2) F	GTTCTCAGCCCAACAATACAAGA	6754872a1
iNOS R	GTGGACGGGTCGATGTCAC	
TNF F	CCCTCACACTCAGATCATCTTCT	7305585a1
TNF R	GCTACGACGTGGGCTACAG	
Arg1 F	CTCCAAGCCAAAGTCCTTAGAG	7106255a1
Arg1 R	AGGAGCTGTCATTAGGGACATC	
CD206 (Mrc1) F	CTCTGTTCAGCTATTGGACGC	6678932a1
CD206 R	CGGAATTTCTGGGATTCAGCTTC	
CCL17 F	TACCATGAGGTCACTTCAGATGC	225735578c1
CCL17 R	GCACTCTCGGCCTACATTGG	

*Each pair is identified with one PrimerBankID, taken from PrimerBank https://pga.mgh.harvard.edu/primerbank/)

2.6 Flow Cytometry

Flow cytometry was conducted at the UCSF Flow Cytometry core on a BD Fortessa instrument. Cells were labelled with antibodies according to manufacturer instructions and the data was analyzed using FlowJo (FlowJo, LLC.). Cell sorting was conducted on a BD FACSAria instrument.

Cell Surface Labeling

1. Spin down 500,000+ cells, 400 g 4 min
2. Resuspend cells in PBS+0.5% FBS
3. Spin cells down again
4. Resuspend cells in PBS+0.5% FBS at 10^6 cells per 100µL
5. Mix 100 µL of cells with 1 µg of unlabeled anti-CD16/32 (FcBlock)
6. Incubate RT, 10 min
7. Add labeling antibody (0.1-1µg) (directly conjugated or biotinylated). For F4/80, use rat IgG2b κ isotype, anti-mouse F4/80 (0.2 µg per 10^6 cells in 100 µL), labelled with APC (Biolegend). Isotype used for control was unlabeled rat IgG2b κ isotype (Biolegend)
8. Incubate on ice 30-60 min
9. Add 200 ng labeled streptavidin if labeling antibody was biotinylated for 10min
10. Add 900 µL PBS+0.5% FBS
11. Spin down cells, 400 g 4 min
12. Resuspend labeled cells in 500 µL PBS+0.5% FBS
13. Read out on FACS (BD Fortessa at Parnassus Flow Cytometry Core, UCSF)

Have neg controls (no Ab, isotype specific non-specific Ab, no-expression-of-target cells)

Fluorescent Protein Cell Analysis

For cells that are fluorescently labeled, cells can be centrifuged and resuspended in D-PBS and analyzed on FACS without any further treatment.

DRAQ7 Staining for Dead Cells

Staining for live/dead cells was done using DRAQ7 (Abcam), which labels dead and apoptotic cells for flow cytometry, using the manufacturer protocols.
